# Bioisosteric Modification of To042: Synthesis and Evaluation of Promising Use‐Dependent Inhibitors of Voltage‐Gated Sodium Channels

**DOI:** 10.1002/cmdc.202100496

**Published:** 2021-10-05

**Authors:** Gualtiero Milani, Maria Maddalena Cavalluzzi, Concetta Altamura, Antonella Santoro, Mariagrazia Perrone, Marilena Muraglia, Nicola Antonio Colabufo, Filomena Corbo, Elisabetta Casalino, Carlo Franchini, Isabella Pisano, Jean‐François Desaphy, Antonio Carrieri, Alessia Carocci, Giovanni Lentini

**Affiliations:** ^1^ Department of Pharmacy – Pharmaceutical Sciences University of Bari Aldo Moro Via E. Orabona 4 70125 Bari Italy; ^2^ Department of Biomedical Sciences and Human Oncology School of Medicine University of Bari Aldo Moro Policlinico Piazza Giulio Cesare 70124 Bari Italy; ^3^ Department of Bioscience, Biotechnology and Biopharmaceutics University of Bari Aldo Moro Via Orabona 4 70125 Bari Italy; ^4^ Department of Veterinary Medicine University of Bari Aldo Moro Via E. Orabona 4 70125 Bari Italy

**Keywords:** bioisosteric replacement, drug discovery, mexiletine, molecular modeling, sodium channel blockers

## Abstract

Three analogues of To042, a tocainide‐related lead compound recently reported for the treatment of myotonia, were synthesized and evaluated in vitro as skeletal muscle sodium channel blockers possibly endowed with enhanced use‐dependent behavior. Patch‐clamp experiments on hNav1.4 expressed in HEK293 cells showed that *N*‐[(naphthalen‐1‐yl)methyl]‐4‐[(2,6‐dimethyl)phenoxy]butan‐2‐amine, the aryloxyalkyl bioisostere of To042, exerted a higher use‐dependent block than To042 thus being able to preferentially block the channels in over‐excited membranes while preserving healthy tissue function. It also showed the lowest active transport across BBB according to the results of P‐glycoprotein (P‐gp) interacting activity evaluation and the highest cytoprotective effect on HeLa cells. Quantum mechanical calculations and dockings gave insights on the most probable conformation of the aryloxyalkyl bioisostere of To042 in solution and the target residues involved in the binding, respectively. Both approaches indicated the conformations that might be adopted in both the unbound and bound state of the ligand. Overall, *N*‐[(naphthalen‐1‐yl)methyl]‐4‐[(2,6‐dimethyl)phenoxy]butan‐2‐amine exhibits an interesting toxico‐pharmacological profile and deserves further investigation.

## Graphical abstract

**Proper neural activity**: A bioisosteric replacement approach allowed the identification of a use‐dependent sodium channel blocker (**17 a**) with low P‐glycoprotein‐mediated active transport across the blood‐brain barrier and a cytoprotective effect on HeLa cells. The target residues involved in binding hNav1.4 were identified by molecular docking studies.

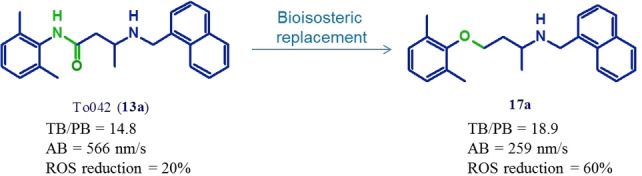

## Introduction

**Figure 1:**
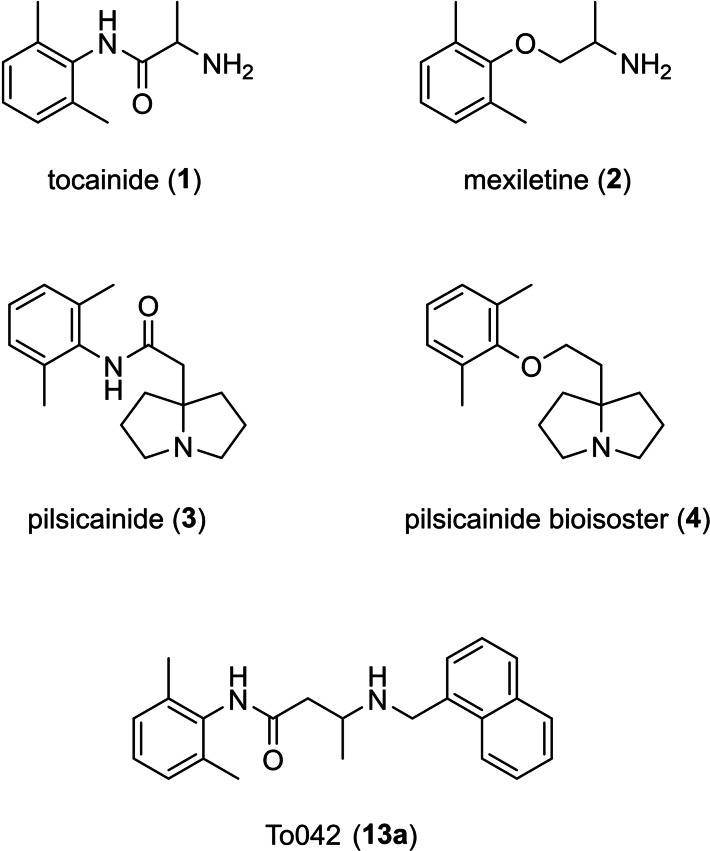
Structures of tocainide (**1**) and its oxymethylene bioisostere mexiletine (**2**), pilsicainide (**3**) and its oxymethylene bioisostere (**4**), and To042 (**13 a**).

Voltage‐gated sodium channels (VGSCs) are transmembrane proteins that mediate the selective influx of sodium ions in excitable cells, thus playing a key role in the regulation of action potential generation and propagation. Alterations in VGSC function or expression can profoundly affect normal cell excitability and lead to a broad range of disorders including epilepsy, pain, muscle disorders, multiple sclerosis, cardiovascular diseases, neurodegeneration, and cancer. In the past decades, a number of interesting small molecule VGSC blockers have been reported as cardiovascular, antiarrhythmic, pain‐relieving and neuroprotective, and antimyotonic agents.^[1]^ Myotonic syndromes are a heterogeneous group of skeletal muscle inherited disorders characterized by enhanced excitability of the muscle fiber that impairs muscle relaxation after activation. The slow relaxation of the muscles causes disabling symptoms such as stiffness and, sometimes, periodic attacks of weakness and pain.[^2^, ^3^, ^4^] Tocainide and mexiletine (Figure 1) are well‐known VGSC blockers belonging to the local anesthetic (LA)‐like drug class, which have long been used off‐label for non‐dystrophic myotonias. However, tocainide was withdrawn from the market in many countries due to harmful agranulocytosis and anemia. After successful randomized clinical trials in myotonia,[^5^, ^6^] mexiletine was recently appointed by EMA as an orphan drug for the treatment of myotonic syndromes.[^7^, ^8^] Mexiletine and tocainide clinical usefulness stems from their ability to inhibit myotonic discharges of action potentials thus favoring muscle relaxation.^[9]^ Both drugs display a mild ability to block sodium channels in a use‐dependent manner, with greater affinity for the channel under conditions of high‐frequency discharge of action potentials, thus mainly acting in pathological rather than physiological excitability conditions.[^10^, ^11^] Although mexiletine is generally well tolerated, lack of tolerability, lack of response, as well as side effects, may occur in a significant number of myotonic patients limiting its usefulness.[^12^, ^13^] As a result of over 20 years of studies that our research group dedicated to the identification of structural determinants of skeletal muscle VCSG block and structure‐activity relationships (SAR),[^14^, ^15^, ^16^, ^17^, ^18^, ^19^, ^20^, ^21^, ^22^, ^23^, ^24^] a new promising candidate drug for myotonia has been identified (To042, Figure 1) which was 120 times more potent than mexiletine in blocking hNav1.4 channels in myotonia‐like cellular conditions, and 100 times more potent in improving muscle stiffness in vivo in the rat model of myotonia.^[14]^ Despite its excellent pharmacodynamic profile, the clinical use of this compound, which represents the *N*‐[(1‐naphthyl)methyl]‐derivative of the tocainide homologue, could be threatened by the presence in its structure of an anilide moiety, a well‐known chemical structural alert.^[25]^ Indeed, the metabolically labile aromatic amides may release the toxic aniline in vivo, as demonstrated for several drugs and drug candidates,^[26]^ with the hazard of bone marrow aplasia,^[27]^ one of the main causes of tocainide withdrawal from the market,^[28]^ and methemoglobinemia.^[29]^ Replacement of susceptible groups with metabolically stable congeners is a common lead optimization procedure in the drug discovery process to obtain safer and less toxic agents,^[25]^ as confirmed by the bioisosteric relationship holding between tocainide (**1**) and its oxymethylene analog mexiletine (**2**), with mexiletine being endowed with a more favorable toxicological profile.^[30]^ A similar improvement was successfully obtained through the same bioisosteric replacement applied to the antiarrhythmic drug pilsicainide (**3**) to obtain the more use‐dependent VGSC blocking agent **4** (Figure 1).^[31]^ On the other hand, mexiletine has already been used in a fragment‐based drug discovery approach^[32]^ where this xylyloxyalkylamine was included as a fragment (i. e. a low‐molecular‐weight compound) in the development of gradually more complex derivatives with increasing potency and maintained ligand efficiency.[^33^, ^34^, ^35^]

All the above findings prompted us to design the oxymethylene bioisostere of To042 (compound **17 a**).

Furthermore, to explore a possible contribution of regioselective interactions of the naphthyl moiety with the channel, we also designed β‐naphthyl analogues of both To042 and its oxymethylene bioisostere (**13 b** and **17 b**, respectively). The synthesis and the use‐dependent hNav1.4 blocking activity of the novel LA‐like drugs are discussed in the present study. Molecular modelling studies based on quantum mechanical calculations and dockings to hNav1.4 structure were also performed to gain insight on the most probable **17 a** conformation and to target residues most likely involved in the binding.

## Results and Discussion

### Chemistry

**Scheme 1:**
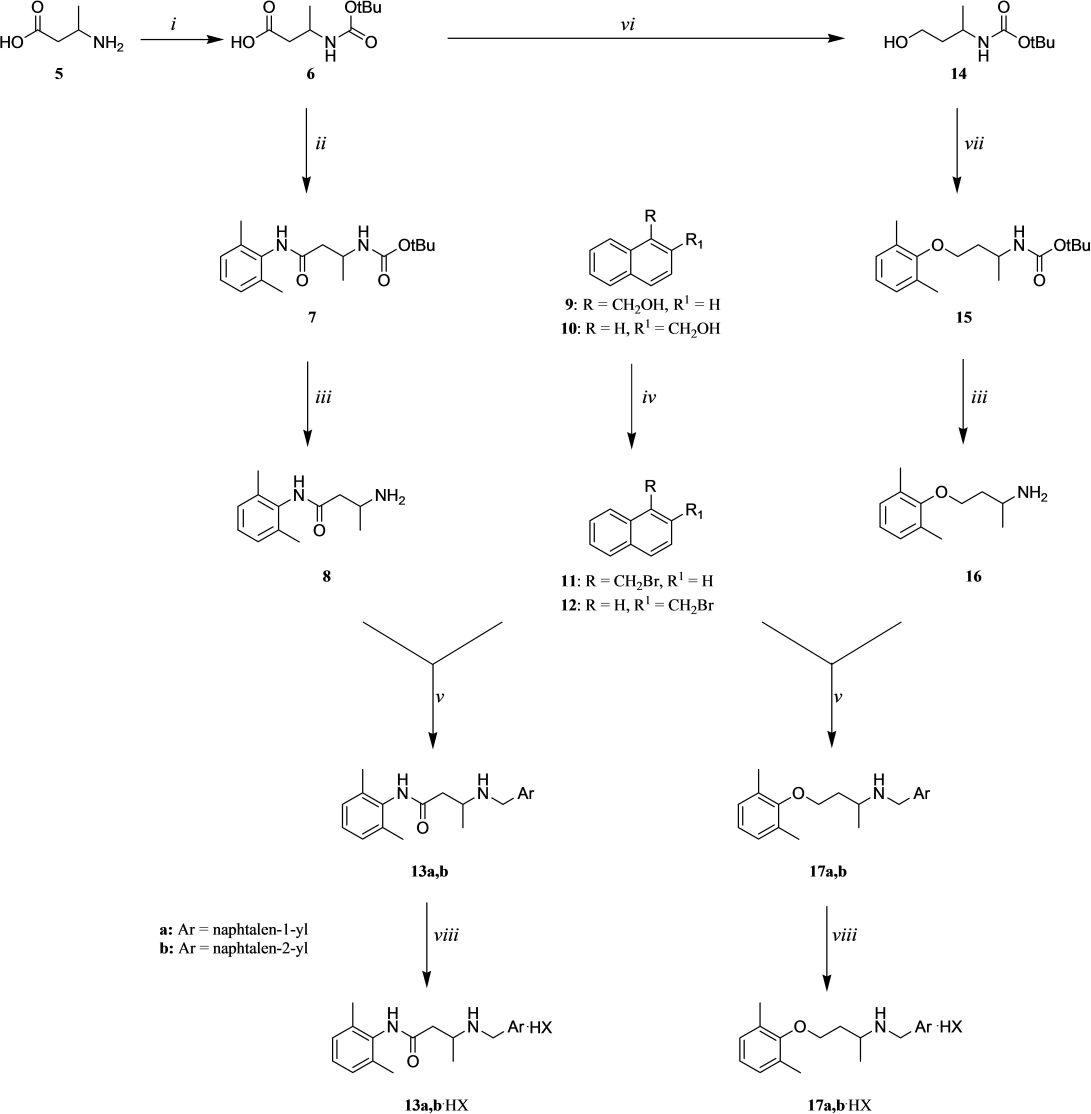
*Reagents and conditions*: i) BOC_2_O, 1 M NaOH, THF, 0 °C, then rt; ii) 2,6‐dimethylaniline, TBTU, DIEA, dry DMF, 0 °C, then rt; iii) CF_3_COOH, CH_2_Cl_2_, rt; iv) PBr_3_, 0 °C, then rt; v) K_2_CO_3_, CH_3_CN, rt; vi) LiAlH_4_, dry THF, 70 °C, then rt; vii) 2,6‐dimethylphenol, TPP, DIAD, dry THF, rt; viii) aq HX.

The target compounds (**13 a,b** and **17 a,b**) were prepared as outlined in Scheme 1. The route starts from the efficient protection of 3‐aminobutyric acid (**5**) with di‐*tert*‐butyl dicarbonate^[36]^ to give the *N*‐Boc protected amino acid **6** which underwent two different synthetic routes. In the first, **6** was reacted with 2,6‐dimethylaniline, in the presence of TBTU as a coupling reagent,^[37]^ to give the intermediate **7**. Deprotection with trifluoroacetic acid gave the tocainide homologue **8** which was reacted with 1‐ or 2‐(bromomethyl)‐naphthalene (**11**, **12**, respectively), in turn obtained starting from the corresponding alcohols (**9, 10**), affording the desired products (**13 a,b**).^[38]^ Compound **13 a** was obtained in a higher overall yield than the previously reported one^[22]^ (48 % vs 9 %, respectively).

In the second route, the *N*‐Boc protected amino acid **6** was reduced to the corresponding alcohol **14** which was subjected to Mitsunobu reaction with 2,6‐dimethylphenol as previously described.^[20]^ Removal of the Boc protecting group gave the mexiletine homologue **16** which was alkylated with 1‐ or 2‐(bromomethyl)naphthalene (**11**, **12**) to afford the desired products (**17 a,b**).^[22]^ All final amines (**13 a,b** and **17 a,b**) were converted into their corresponding hydrohalides before undergoing biological assays.

**Scheme 2:**
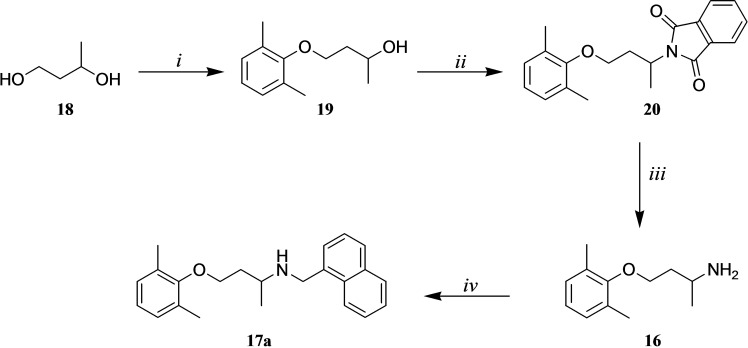
*Reagents and conditions*: i) 2,6‐dimethylphenol, TPP, DIAD, dry THF, rt; ii) phthalimide, TPP, DIAD, dry THF, rt; iii) aq N_2_H_4_ 55 %, glacial AcOH, MeOH, reflux; iv) 1‐(bromomethyl)naphthalene (**11**), K_2_CO_3_, CH_3_CN, rt.

Following a second route to obtain the amine **17 a** (Scheme 2), 1,3‐butanediol (**18**) was subjected to Mitsunobu reaction with 2,6‐dimethylphenol to give the alcohol **19** which underwent a further Mitsunobu reaction with phthalimide. Then, the phthalimido intermediate **20** was deprotected by hydrazinolysis^[39]^ to give **16** which was alkylated using 1‐(bromomethyl)‐naphthalene (**11**) to give **17 a**. The desired product was obtained in an overall yield slightly lower than the one reached in Scheme 1.

### Biological results

**Figure 2:**
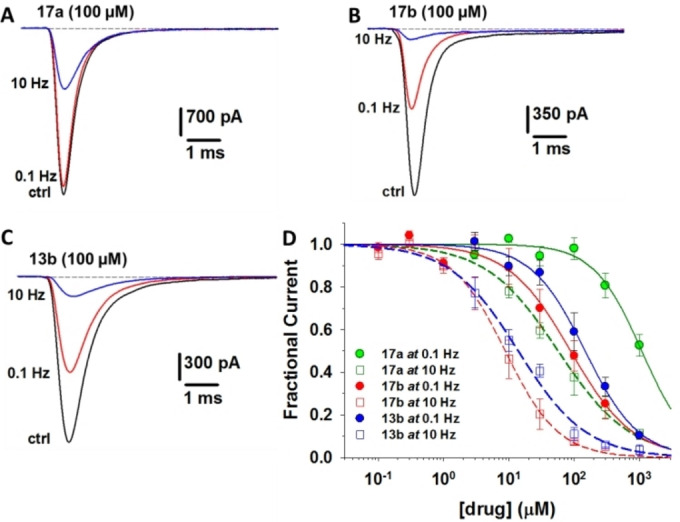
Effects of the synthesized exploratory compounds (**13 b, 17 a, 17 b**) on hNav1.4 sodium currents. **A**–**C**) Representative sodium currents recorded in HEK293T cells expressing hNav1.4 channels in control conditions (CTRL) and during application of 100 μM exploratory compounds at 0.1 or 10 Hz stimulation frequency. **D**) Concentration‐response curves of exploratory compounds at 0.1 and 10 Hz stimulation frequencies. Data points are reported as mean values ± SEM from 4–12 cells.

The newly synthesized compounds were tested on hNav1.4 expressed in HEK293 cells, using patch‐clamp technique (Table 1 and Figure 2). The obtained data showed that To042 (**13 a**) was the most potent hNav1.4 blocker both in phasic and tonic block, with IC_50_ values of 12 and 0.81 μM for phasic and tonic block, respectively. Nevertheless, its mexiletine bioisostere **17 a** exerted a use‐dependent behavior greater than To042, with its ratio IC_50 tonic block_/IC_50 phasic block_ (TB/PB) value being not only greater than that found for To042 but also the highest among all the tested compounds (TB/PB=18.9, Table 1). Therefore, despite a 6‐fold higher IC_50_ value at 10 Hz (57 μM for To042 vs 0.81 μM for **17 a**), the bioisostere **17 a** showed a greater ability to block channels in over‐excited membranes and is thus expected to preserve healthy tissue functions better than To042. Furthermore, the mexiletine analogue **17 a** should be devoid of the To042 metabolic liability resulting from the presence of the xylidide moiety in To042 structure; as its parent compound tocainide, **13 a** might undergo in vivo hydrolysis of the amide bond, thus leading to the formation of toxic xylidine. Therefore, the bioisostere **17 a** should be a safer drug due to both use‐dependence of its VGSC blocking activity and reduced metabolic liability. On the other hand, it would be safer even than mexiletine, the only clinically used compound among those tested herein: **17 a** showed a greater use‐dependence with its TB/PB being three times higher than that of mexiletine as a consequence of its four‐fold reduced tonic block potency (IC_50_ values 1080±150 and 256±25, respectively) linked to similar potency in the phasic block. As regards the beta regioisomers (**13 b**, **17 b**), a peculiar regioselective behavior has been observed within the two bioisosteric series of compounds.

**Table 1:** Half‐maximal concentrations (IC_50_) and slope factors (nH) for tonic block and phasic block of hNav1.4 sodium currents for mexiletine, tocainide, and their analogues.^[a]^

Compd	Tonic Block IC_50_ at 0.1 Hz (μM)	nH	Phasic Block IC_50_ at 10 Hz (μM)	nH	TB/PB
mex^[b]^	256±25	1.2±0.1	46±5	0.9±0.1	5.6
toc^[c]^	700±70	1.0±0.1	182±24	1.1±0.2	3.8
**13 a** ^[d]^	12±1	1.3±0.1	0.81±0.06	0.8±0.1	14.8
**13 b**	147±12	1.1±0.1	14±1.8	0.8±0.1	10.5
**17 a**	1080±150	1.2±0.2	57±8	0.8±0.1	18.9
**17 b**	88±11	0.9±0.1	8.8±0.6	1.1±0.1	10

[a] Fit parameter values are given with standard error of the fit. [b] Mexiletine.^[44]^ [c] Tocainide.^[45]^ [d] Ref. [14].

In fact, the beta isomer among the tocainide analogues was less potent than the corresponding alpha isomer, regardless of the applied stimulation frequencies (0.1 and 10 Hz), while the opposite was true for the mexiletine analogues. It is worth noting that **17 b** was more potent and displayed higher use‐dependency of action than its parent compound mexiletine. The use‐dependent behavior of sodium channel blockers, including mexiletine, is generally attributed to the higher affinity of the drugs for open and/or fast‐inactivated channels compared to closed channels.[^40^, ^41^] Although observed at rather high mexiletine concentrations,^[42]^ enhancement of slow inactivation might also be of interest for drug efficacy in myotonia.^[43]^ Further studies are warranted to verify whether chemical maneuvers may affect the molecular mechanisms of VGSC inhibition.

Since the central nervous system (CNS) side effects of both mexiletine and tocainide are well‐known, the ability of the tested compounds to cross the blood‐brain barrier (BBB) was evaluated. Their apparent permeability (Papp) across epithelial colorectal adenocarcinoma (Caco‐2) cell monolayer overexpressing human P‐gp in both the basolateral–apical (BA) and apical–basolateral (AB) directions was measured (Table 2). This assay is commonly referred to as a reliable predictor of BBB penetration.^[46]^ Oddly, any of the compounds appeared able to cross the BBB since their BA/AB value is >2, as expected for good P‐gp substrates. However, these results are inconsistent with the well‐known human neurotoxicity of the two parent compounds. Actually, a more careful analysis of the obtained AB values, with the mexiletine analogue **17 a** showing the lowest value among all the tested compounds (AB=259 nm/s), indicates the least P‐gp active transport across BBB to enter the CNS for this compound. On the contrary, not only To042 showed an AB value about 2.5‐fold higher than that of **17 a** but its active transport across BBB should be the highest of all the newly synthesized compounds and the closest to that of mexiletine, whose CNS toxicity is a well‐known side effect. Therefore, the sum of these findings identifies **17 a** as the best candidate for more in‐depth studies as a possible antimyotonic agent acting peripherically.

**Table 2:** Apparent permeability of mexiletine, tocainide, and their analogues across Caco‐2 cells monolayers overexpressing human P‐gp in both the Basolateral‐to‐Apical (BA) and Apical‐to‐Basolateral (AB) directions.

Compd.	P_app_ (nm/s)		
	BA	AB	BA/AB
mexiletine	2430	642	3.8
tocainide	1965	496	3.9
**13 a**	2234	566	4.1
**13 b**	2157	438	4.9
**17 a**	1802	259	7.1
**17 b**	1993	496	4.0

**Figure 3:**
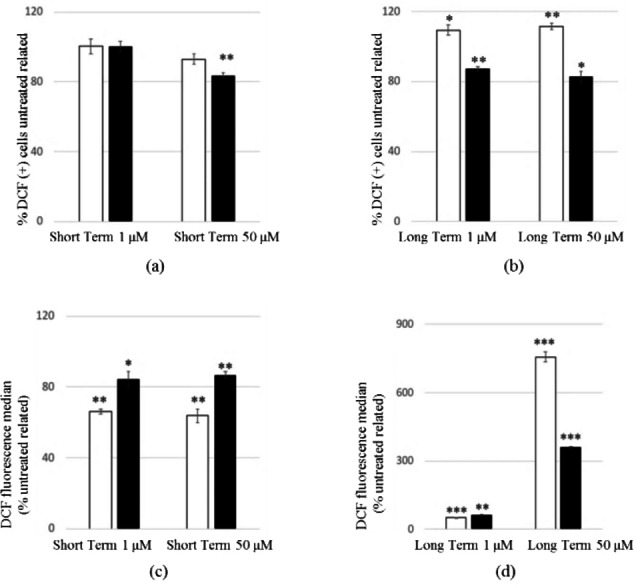
Effect of **13 a** and **17 a** on ROS production of HeLa cells. HeLa cells were exposed to 1 and 50 μM of **13 a** (empty bar) and **17 a** (black bar) at 6 h (Short Term) (a,c) and 24 h (Long Term) (b,d). After washing with HBSS, cells were stained with DCF and the percentage of ROS producing cells (a,b) (expressed as % DCF (+) cells untreated related) and the intracellular ROS levels (c,d) (expressed as % DCF fluorescence median untreated related) were detected by flow cyotometry analysis. Data are expressed as the mean ± standard deviation (error bars) from at least three independent experiments. The data were analyzed by Student's *t* test; *p < 0.05, **p < 0.01, ***p < 0.001 to the untreated control.

**Figure 4:**
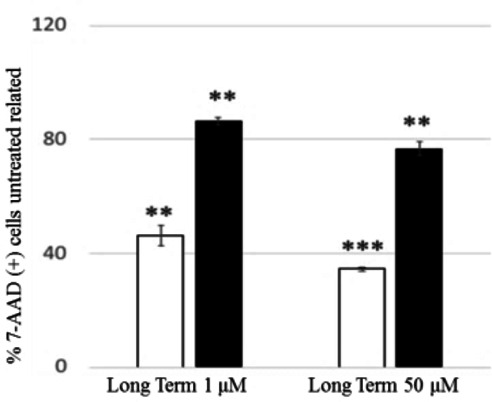
Effect of **13 a** and **17 a** on viability of HeLa cells. HeLa cells were exposed to 1 and 50 μM of **13 a** (empty bar) and **17 a** (black bar) at 24 h (Long Term). After washing with HBSS, cells were co‐stained with DCF and 7‐AAD and the percentages of damaged cells (expressed as percentage of 7‐AAD positive cells untreated related) were detected by flow cyotometry analysis. Data are expressed as the mean±standard deviation (error bars) from at least three independent experiments. The data were analysed by Student's *t* test; *p < 0.05, **p < 0.01, ***p < 0.001 to the untreated control.

The myotoxicity of **13 a** had been previously evaluated by a cell viability test on C2 C12 murine skeletal muscle cells^[14]^ and a significant reduction in cell viability between 10 and 100 μM was observed. To evaluate the potential cytotoxic effects of the two most interesting compounds (**13 a** and **17 a**) against human cell lines, the human cervical epithelioid carcinoma (HeLa) cell line was selected based on our previously reported tocainide data.^[45]^ To assess oxidative damage potential, the flow cytometry analysis was performed using the 2′,7′‐dichlorodihydrofluorescein (DCF) assay. Furthermore, since mitochondrial dysfunction induced by aniline^[47]^ and local anesthetic drugs^[48]^ is known, a more in‐depth evaluation of mitochondrial oxidative stress was carried out using the dihydrorhodamine (DHR) assay. As shown in Figure 3a, no significant effects were observed in terms of percentage of ROS producing cells related to untreated cells when the cells were exposed for 6 h to 1 μM concentration of both drugs. Conversely, a significant cytoprotective effect was observed at 50 μM only for **17 a**. Moreover, a clearly different behavior of the two compounds after 24 h, regardless of concentrations, highlights the prominent cytoprotective effect of **17 a** compared to **13 a** (Figure 3b). These data are confirmed by panel 2d which shows approximately 8‐ and 3‐fold higher ROS production by 50 μM **13 a** and 50 μM **17 a**, respectively, related to untreated cells after 24 h. Given the significant increase in ROS production observed mostly for compound **13 a** at 50 μM concentration passing from short (Figure 3c) to long term exposure (Figure 3d), it is reasonable to assume that the greater oxidative stress induced over time by **13 a** might arise from time‐dependent metabolic processes, conceivably leading to toxic metabolites. However, the assay with 7‐AAD demonstrated that cellular viability is not affected within 24 h (Figure 4).

**Figure 5:**
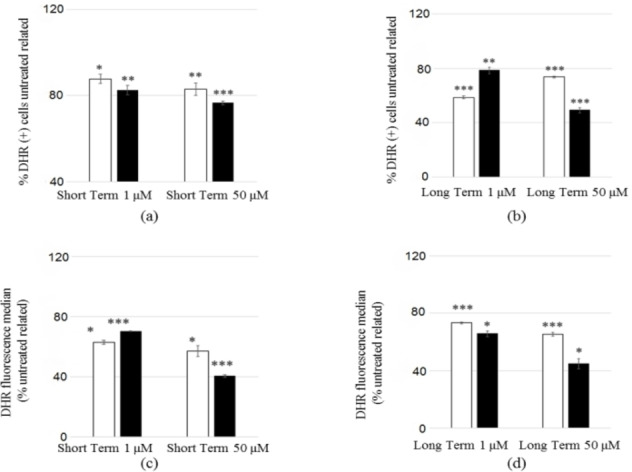
Effect of **13 a** and **17 a** on mitochondrial ROS production of HeLa cells. HeLa cells were exposed to 1 and 50 μM of **13 a** (empty bar) and **17 a** (black bar) at 6 h (Short Term) (a,c) and 24 h (Long Term) (b,d). After washing with HBSS, cells were stained with DHR and the percentage of ROS producing cells (a,b) (expressed as % DHR (+) cells untreated related) and the mitochondrial ROS levels (c,d) (expressed as % DHR fluorescence median untreated related) were detected by flow cyotometry analysis. Data are expressed as the mean ± standard deviation (error bars) from at least three independent experiments. The data were analysed by Student's *t* test; *p < 0.05, **p < 0.01, ***p < 0.001 to the untreated control.

Interestingly, when mitochondrial ROS production was evaluated (Figure 5), both compounds showed cytoprotective effects, with the number of ROS producing cells (Figure 5a and 5b) and the levels of produced ROS (Figure 5c and 5d) always being lower than control, regardless of the exposure time (6 and 24 h) and the tested concentrations (1 and 50 μM).

It is worth noting that the oxymethylene bioisostere **17 a** at 50 μM concentration showed the lower percentage of ROS producing cells and also the lower ROS levels. In particular, focusing our attention on the results obtained at 50 μM for **17 a** and 1 μM for **13 a**, these values being close to the concentrations responsible for the 50 % inhibition of hNav1.4 channels in the phasic block (IC_50_ 57±8 μM and 0.81±0.06 μM for **17 a** and **13 a**, respectively; Table 1), we can state that **17 a** preserves mitochondrial functionality more than **13 a** when their respective active concentrations are considered. Since mitochondrial function is a key component of skeletal muscle health,^[49]^ the obtained results throw light on oxymethylene bioisostere **17 a**.

### Molecular modelling studies

A proper mechanistic understanding underlying the phasic and tonic blocks has long been hampered by a paucity of information on the molecular architecture of voltage‐gated ion channels. The studies on this issue have been recently boosted by the determination of the cryo‐EM structure of hNav1.4 by Pan et al.^[50]^ This knowledge led us to gain fresh insights into the pharmacological profile of some of the ligands presented herein by exploiting the chemical interaction pattern with the relative molecular target. To address this topic, docking simulations were run on To42 (**13 a**), the most potent blocking agent reported herein.

To start with, a conformational analysis was run on the protonated model of To42 in the gaseous phase following our previously developed procedures.[^51^, ^52^] As expected for a relatively flexible molecule, a high number of conformers (22) were found within a window of 3 kcal/mol above the global minimum conformation energy. However, all conformers shared three main features:

1. the planes of the xylyl ring and the amide group were orthogonal;
2. the conformations were stabilized by an intramolecular hydrogen bond linking the amide carbonyl group and the protonated head;
3. the protonated nitrogen atom was roughly on the same plane of the amide group.

**Figure 6:**
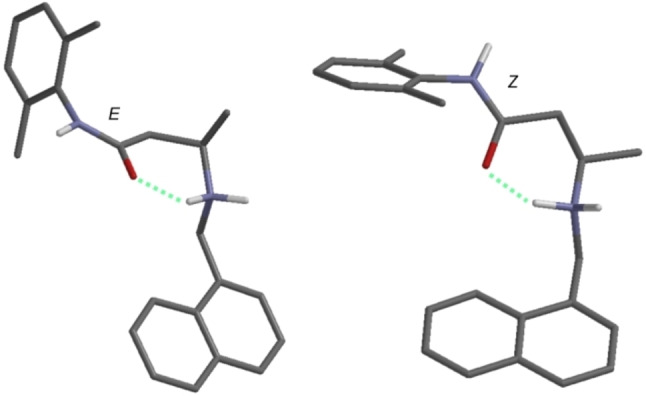
Representative stable conformations of the two *sp* (i. e., *E*; left half of the panel)/*ap* (i. e., *Z*, right half of the panel) clusters found as a result of the conformational analysis run on To42 (**13 a**, DFT B3LYP/6‐31G*//DFT B3LYP/6‐31G*); for the sake of simplicity, only relevant hydrogen atoms were shown, with the dotted lines indicating intramolecular hydrogen bonds.

The above features were conserved also when running the same study in MeOH as a solvent. The 22 conformers could be clustered in two groups differing in the *sp* (i. e., *E*)/*ap* (i. e., *Z*) orientation of the amide NH and CO groups. The two most stable conformers representative of each cluster are reported in Figure 6.

Both conformations were true conformers (no imaginary IR frequencies, DFT EDF2/6‐31G*//DFT B3LYP/6‐31G*). The reliability of our analysis was checked calculating the ^1^H NMR frequencies for each of the whole set of conformers (DFT EDF2/6‐31G*//DFT B3LYP/6‐31G*) and comparing the Boltzmann factor weighted resonance frequencies with those experimentally obtained. Since a good agreement was found (see Table S1), the above reported geometrical features 1–3 were considered as a benchmark for the conformations found in the following docking simulation study.

**Figure 7:**
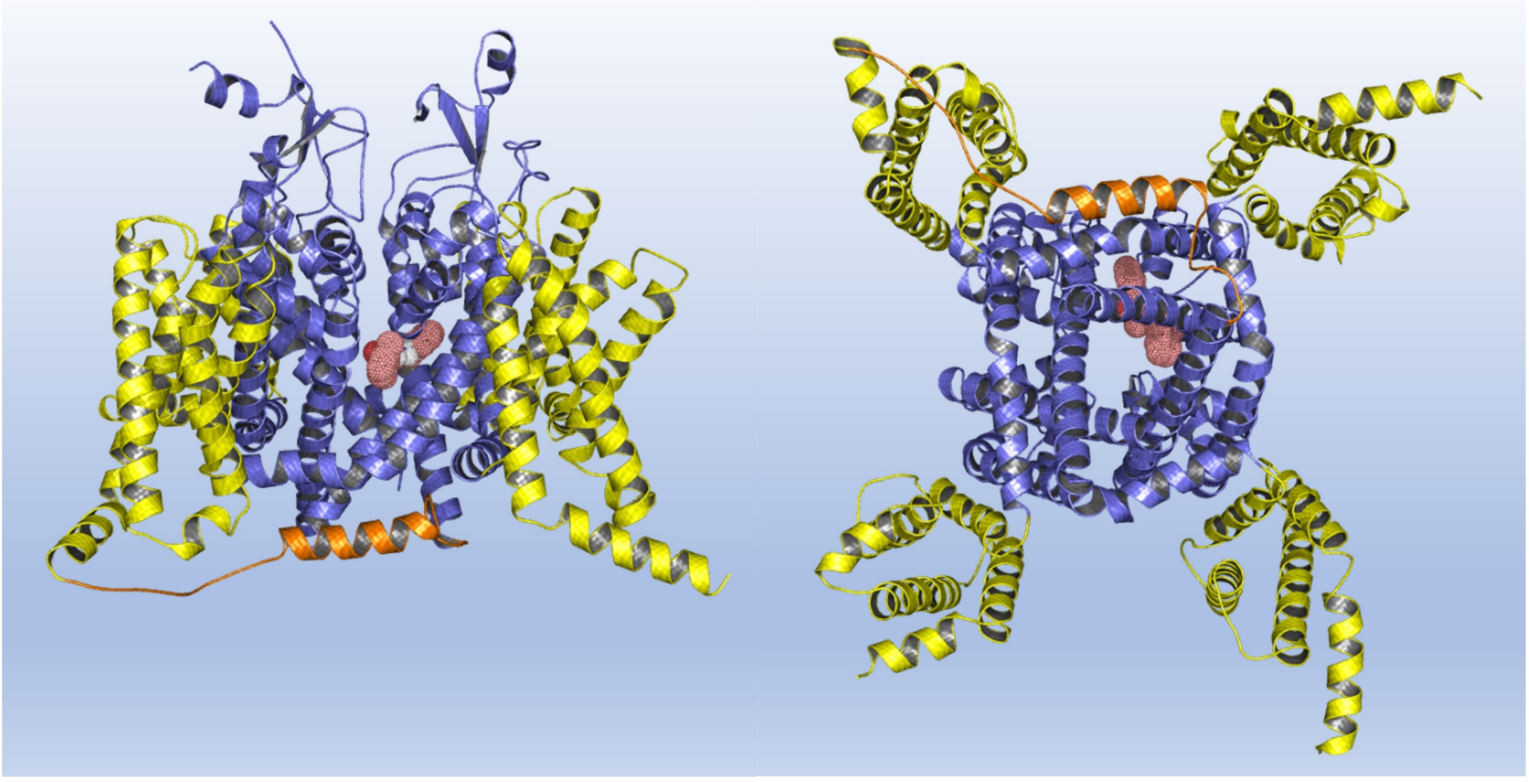
Docking of **13 a** to hNav1.4: side (left) and bottom (right) view of intracellular gate and the cavity. The VSDs, the pore and the linker accommodating the IFM motif are depicted in yellow, slate and orange respectively, while **13 a** is shown as dots.

The overall structure of the sodium channel is characterized by four nonidentical repeats (domains I to IV), each comprising six transmembrane helical segments. The fourth segment of each domain constitutes a voltage sensor. The symmetrical arrangement of the four domains defines a central pore, which is the permeation pathway for sodium ions. Conformational arrangements of the voltage sensors allow the transitions between resting, activated, and inactivated states.^[53]^ The DEKA residues located at the outermost mouth of the pore form the selectivity filter, responsible for the specific Na^+^ permeation.[^54^, ^55^] An IFM motif, part of the linker connecting DIII and DIV, is thought to represent the region where ligands could exert their channel blockade probably by an allosteric mechanism.[^56^, ^57^] The local anesthetic‐like ligands are postulated to bind amino acids of the 6^th^ segments of domains inside the pore.[^58^, ^59^, ^60^] As long as this evidence is concerned, we tested the complementarity of the **13 a** molecular scaffold with the three‐dimensional structure of hNav1.4 by means of docking simulations. As can be seen from Figure 7, the binding agent fits well into a deep gorge of the channel, characterized mainly by the presence of hydrophobic and aromatic residues, one of which, namely Phe1586, is essential for binding, as confirmed by site‐directed mutagenesis experiments showing that this same residue is critical for the binding of mexiletine analogs to the channel, as well as for other local anesthetic‐like drugs including **13 a**.[^14^, ^61^]

In detail, **13 a** is completely buried in the pore and interacts mainly with the transmembrane helix 6 residues of repeat I (position 425–463) and repeat IV (position 1522–1534) and simultaneously with one of the four intracellular pore‐forming helices (position 1522–1534).

**Figure 8:**
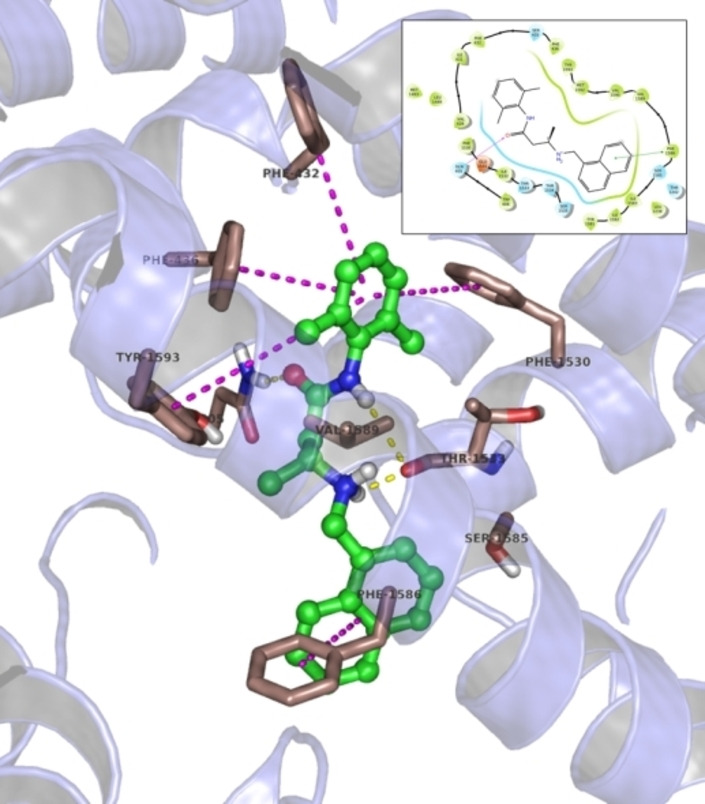
Detailed view of the docking of **13 a** to hNav1.4: the ligand is shown as ball and sticks in orange. Hydrogen bonds are shown in yellow, π‐π stacking in magenta.

Several types of interactions enhance the binding of the blocker to the ion channel: the molecular *lipole* characterized by aromatic (dimethylphenyl and α‐naphthyl) pendants anchor **13 a** with edge‐to‐face stacking to the aromatic cluster that includes Phe432, Phe436, Phe1530, Tyr1593, and face‐to‐face with Phe1586 (Figure 8). As mentioned earlier, and not coincidentally, the latest two residues are advantageous in their interaction with **13 a**, as the mutation to cysteine impairs ligand binding compared to WT hNav1.4.[^14^, ^61^] The basic protonated and polar core of the molecule further enhances the strength of channel binding with more than one hydrogen bond involving the backbone of Thr1533 and the amide terminal of Gln405. Overall, this molecular aggregate exhibits a favorable binding free energy of −8.40 kcal/mol and a ligand efficiency of −0.323 kcal/mol‐atom.

**Figure 9:**
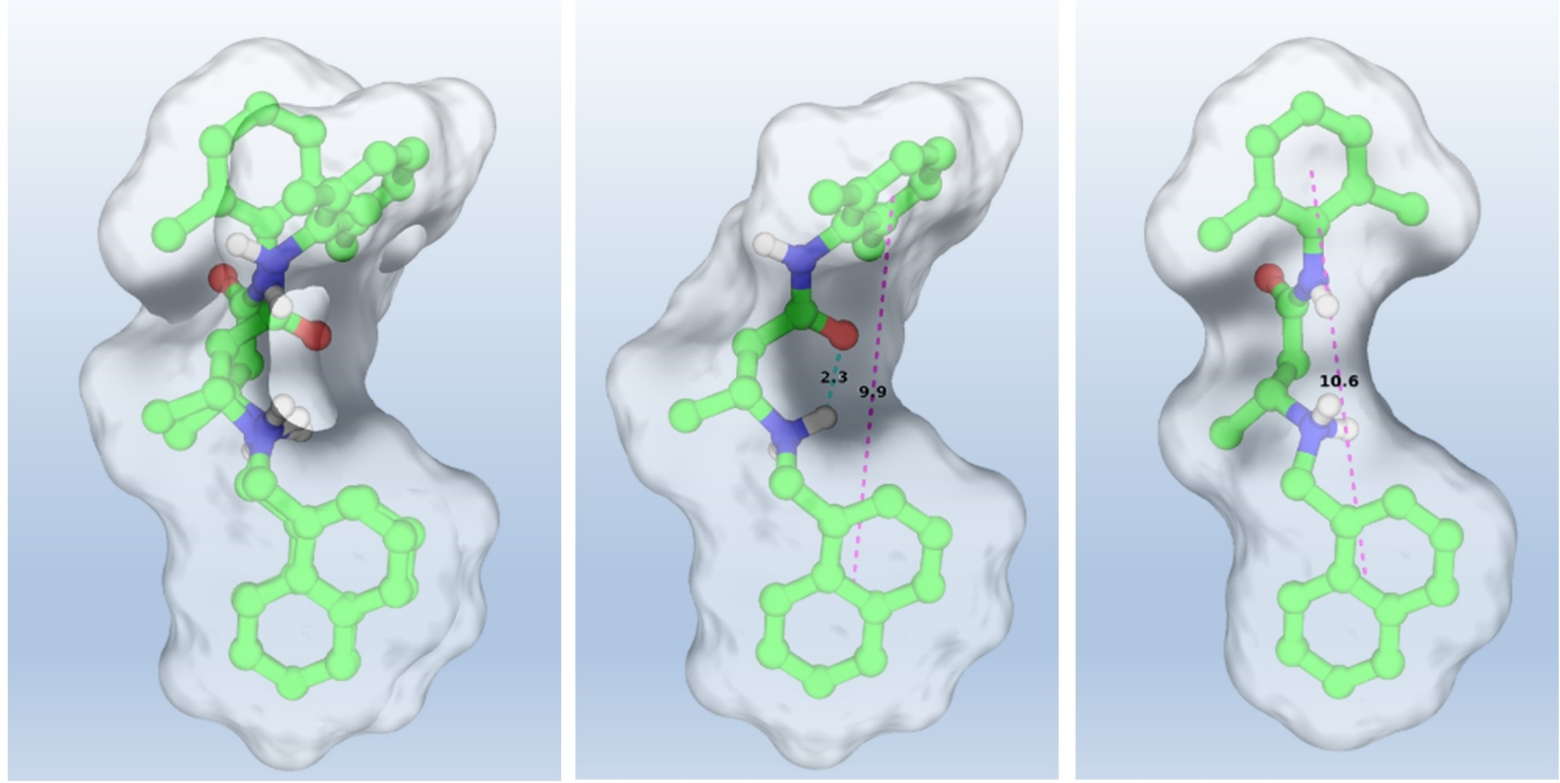
Molecular superposition (left) of the quantum chemical analysis (center) *vs* docking conformation (right) of **13 a**. The intramolecular hydrogen bond and distances between the xylyl and naphthyl rings are shown in cyan and magenta, respectively.

It should be mentioned that the binding site, although not part of the IFM motif, is essentially very close, and it might then represent an alternative, and also allosteric, binding site for molecules acting as ion channel blockers. Finally, it is further interesting to note that this bioactive conformation of **13 a** conserves two out of three main geometrical features (1–3) found as an outcome of the previous quantum chemical analysis, with the intramolecular hydrogen bond being sacrificed in favor of three hydrogen bonds with the binding site. This might perceive from the shape‐based matching between the two conformations as reported in Figure 9, which is indeed afforded by a Tanimoto similarity coefficient equal to 1.087.

## Conclusion

The mexiletine analogue **17 a**, although less potent than the corresponding tocainide bioisostere **13 a**, takes advantage from greater use‐dependence, a conceivably lower neurotoxicity, greater mitochondrial cytoprotective effects against oxidative stress, as well as a greater metabolic stability. Furthermore, with respect to mexiletine, the currently first choice drug in myotonia,^[14]^ it showed a similar potency in producing a phasic block and even greater use‐dependence. Therefore, the use of the mexiletine analogue **17 a** in lieu of mexiletine could be envisaged provided its thorough ADMET profile is favorable.

## Experimental Section

### Chemistry

Chemicals were purchased from Sigma‐Aldrich or Alpha‐Aesar. Yields refer to purified products and were not optimized. The structures of the compounds were confirmed by routine spectrometric and spectroscopic analyses. Only spectra for compounds not previously described are given. Melting points were determined on a Gallenkamp apparatus in open glass capillary tubes and are uncorrected. ^1^H NMR and ^13^C NMR spectra were recorded on either a Varian VX Mercury spectrometer operating at 300 and 75 MHz for ^1^H and ^13^C, respectively, or an AGILENT 500 MHz operating at 500 and 125 MHz for ^1^H and ^13^C, respectively, using CDCl_3_ as solvent, unless otherwise indicated. Chemical shifts (δ) are reported in ppm relative to the residual non‐deuterated solvent resonance: CDCl_3_, δ=7.26 (^1^H NMR) and δ=77.3 (^13^C NMR) as internal reference. Coupling constants (*J*) are given in Hz. Gas chromatography (GC)/mass spectroscopy (MS) was performed on a Hewlett‐Packard 6890–5973 MSD at low resolution. HRMS analyses were performed using a Bruker microTOF QII mass spectrometer (Bruker, Bremen, Germany) equipped with ESI operating in positive ion mode. Elemental analyses were performed on a Eurovector Euro EA 3000 analyzer and the data for C, H, N were within ±0.4 of theoretical values. Chromatographic separations were performed on silica gel columns by column chromatography on silica gel (Kieselgel 60, 0.040–0.063 mm, Merck, Darmstadt, Germany). TLC analyses were performed on precoated silica gel on aluminum sheets (Kieselgel 60 F_254_, Merck).

#### 3‐[(*tert*‐Butoxycarbonyl)amino]butyric acid (6)

A solution of Boc_2_O (5.08 g, 23.3 mmol) in THF (14 mL) was added dropwise to a solution of 3‐aminobutyric acid (**5**) (2.0 g, 19.4 mmol) and 1 M NaOH (0.776 g, 19.4 mmol) in 40 ml di THF and the mixture was stirred at room temperature for 60 h. After filtration, the solvent was removed under vacuum and the aqueous phase was acidified with 2 M HCl and extracted with EtOAc. The combined organic layers were dried (Na_2_SO_4_) and concentrated under vacuum to give 3.82 g (97 %) of a yellowish oil which was crystallized from EtOAc/petroleum ether giving **6** (75 %) as white crystals: mp 102–104 °C; ^1^H NMR (500 MHz): *δ* 1.23 (d, *J*=6.7 Hz, 3H, C*H*_3_CH), 1.44 (s, 9H, *t*‐Bu), 2.44–2.62 (m, 2H, C*H*_2_), 4.04 (apparent br s, 1H, C*H*), 4.96 (br s, 1H, N*H*); ^13^C NMR (125 MHz): *δ* 20.4 (1 C), 28.3 (3 C), 40.6 (1 C), 43.3 (1 C), 79.6 (1 C), 155.2 (1 C), 176.4 (1 C); GC‐MS (70 eV) *m/z* (%) 188 (M^+^ −15, <1), 57 (100).

#### *tert*‐Butyl [4‐(2,6‐dimethylanilino)‐4‐oxobutan‐2‐yl]carbamate (7)

To a solution of 3‐[(*tert*‐butoxycarbonyl)amino]butyric acid (**6**) (0.200 g, 0.99 mmol) in dry DMF (5 mL), DIEA (0.383 g, 2.97 mmol) was added at 0 °C under argon atmosphere and the solution was stirred for 10 min at 0 °C. TBTU (0.478 g, 1.49 mmol) was added to the solution and the reaction mixture was stirred at 0 °C for 20 min and at room temperature for 3 h. Freshly distilled 2,6‐dimethylaniline (0.180 g, 1.49 mmol) was added and the mixture was stirred at room temperature for 16 h. After evaporation of the solvent, without work‐up, the desired product **7** was obtained through crystallization from MeOH/EtOAc as white crystals (96 %): mp 187–189 °C; ^1^H NMR (500 MHz, DMSO‐*d*_6_): *δ* 1.09 (d, *J*=6.4 Hz, 3H, C*H*_3_), 1.36 (s, 9H, *t*‐Bu), 2.09 (s, 6H, C*H*_3_Ar), 2.33 (dd, *J*=13.7, 7.3 Hz, 1H, C*H*H), 2.46 (dd overlapping DMSO‐*d*_6,_ *J*=13.7, 6.8 Hz, 1H, CH*H*), 3.85–3.95 (m, 1H, C*H*), 6.73 (d, *J=*7.3 Hz, 1H, N*H*CH), 7.02 (apparent s, 3H, Ar), 9.20 (s, 1H, N*H*Ar); ^13^C NMR (125 MHz, DMSO‐*d*_6_): *δ* 18.6 (2 C), 21.2 (1 C), 28.7 (3 C), 42.8 (1 C), 44.4 (1 C), 78.0 (1 C), 126.8 (1 C), 128.0 (2 C), 135.5 (2 C), 135.6 (1 C), 155.2 (1 C), 169.3 (1 C); GC‐MS (70 eV) *m/z* (%) 306 (M^+^, <1), 121 (100); Anal. Calcd for C_17_H_26_N_2_O_3_⋅1.2H_2_O: C, 62.25; H, 8.73; N, 8.54. Found: C, 62.42; H, 8.68; N, 8.86.

#### 3‐Amino‐*N*‐(2,6‐dimethylphenyl)butanamide (8)

A solution of *tert*‐butyl [4‐(2,6‐dimethylanilino)‐4‐oxobutan‐2‐yl]carbamate (**7**) (0.400 g, 1.31 mmol) in trifluoracetic acid (1.26 mL) and CH_2_Cl_2_ (5 mL) was stirred at room temperature for 4 h. The solvent was evaporated in vacuo and the residue was taken up with EtOAc and extracted with 2 M HCl; then the aqueous phase was made alkaline and extracted with EtOAc. The combined organic layers were washed with H_2_O, dried over Na_2_SO_4_ and concentrated under vacuum to give 0.267 g of a yellowish oil (99 %). Spectrometric and spectroscopic data were in agreement with the literature.^[22]^

#### 1‐(Bromomethyl)naphthalene (11)

Phosphorus tribromide (0.66 mL, 6.96 mmol) was added dropwise to 1‐naphthalenemethanol (**9**) (1 g, 6.33 mmol) and the reaction mixture was stirred at 0 °C for 2 h, then at room temperature for 18 h. The mixture was poured on ice and extracted with EtOAc. The combined organic layers were dried over Na_2_SO_4_ and concentrated under vacuum to give 1.19 g (85 %) of a brown oil. Spectrometric and spectroscopic data were in agreement with those reported in the literature.^[22]^

#### 2‐(Bromomethyl)naphthalene (12)

Prepared as reported for **11** starting from **10**. Brown solid (87 %): mp 53–55 °C. Spectrometric and spectroscopic data were in agreement with those reported in the literature.[^62^, ^63^]

#### *N*‐(2,6‐Dimethylphenyl)‐3‐[(naphthalen‐1‐ylmethyl)amino]butanamide (13 a)

A solution of 1‐(bromomethyl)naphthalene (**11**) (0.345 g, 1.56 mmol) in acetonitrile (9 mL) was added dropwise to a magnetically stirred solution of 3‐amino‐*N*‐(2,6‐dimethylphenyl)butanamide (**8**) (0.267 g, 1.3 mmol) and K_2_CO_3_ (0.359 g, 1.56 mmol) in acetonitrile (20 mL). The solution was stirred at room temperature for 20 h. After evaporation of the solvent, the residue was taken up with EtOAc and washed with brine. The organic layer was dried (Na_2_SO_4_) and evaporated under vacuum. The residue was purified by silica gel column chromatography (EtOAc/hexane, 1 : 1) to give 0.256 g (57 %) of **13 a** as a yellowish oil: GC‐MS (70 eV) *m/z* (%) 346 (M^+^, 1), 156 (100); spectroscopic data were in agreement with those reported in the literature.^[22]^ The corresponding hydrochloride (**13 a**⋅HCl) was obtained dissolving the free amine in 1 mL of 2 M HCl and then azeotropically removing water (abs EtOH/toluene) affording a solid which was recrystallized from abs EtOH/Et_2_O to give 0.359 g of white crystals (80 %): mp 198–200 °C. Spectrometric and spectroscopic data were in agreement with those reported in the literature. Anal. Calcd for C_23_H_26_N_2_O⋅HCl⋅0.5H_2_O: C, 70.48; H, 7.20; N, 7.15; Found: C, 70.86; H, 7.02; N, 7.03.

#### *N*‐(2,6‐Dimethylphenyl)‐3‐[(naphthalen‐2‐ylmethyl)amino]butanamide (13 b)

Prepared as reported for **13 a** starting from **12**. Yellowish oil (39 %): GC‐MS (70 eV) *m/z* (%) 205 (M^+^ – 141, 8), 141 (100); ^1^H NMR (300 MHz): *δ* 1.37 (d, *J*=6.4 Hz, 3H, C*H*_3_CH), 1.74 (br s, exch with D_2_O, 1H, N*H*CH), 2.18 (s, 6H, C*H*_3_Ar), 2.45 (dd, *J*=16.4, 7.6 Hz, 1H, C*H*HCH), 2.68 (dd, *J*=16.4, 3.5 Hz, 1H, CH*H*CH), 3.20–3.35 (m, 1H, C*H*), 4.09 (d, *J*=7.0 Hz, 1H, C*H*HNH), 4.14 (d, *J*=7.0 Hz, 1H, CH*H*NH), 7.06 (apparent s, 3H, Ar), 7.38 (d, *J*=8.8 Hz, 1H, Ar), 7.40–7.50 (m, 2H, Ar), 7.60–7.72 (m, 4H, Ar), 9.80 (br s, exch with D_2_O, 1H, N*H*Ar). **13 b**⋅HCl was prepared as reported for **13 a**⋅HCl and was recrystallized from abs EtOH/Et_2_O (56 %): mp 199–201 °C; ^1^H NMR (300 MHz, CD_3_OD): *δ* 1.55 (d, *J*=6.4 Hz, 3H, C*H*_3_CH), 2.20 (s, 6H, C*H*_3_Ar), 2.96 (dd, *J*=5.9, 1.8 Hz, 2H, C*H*_2_CH), 3.75–3.85 (m, 1H, C*H*), 4.49 (s, 2H, C*H*_2_NH), 7.05–7.15 (m, 3H, Ar), 7.54–7.57 (m, 2H, Ar), 7.6 (dd, *J*=8.2, 1.8 Hz, 1H, Ar), 7.86–7.94 (m, 2H, Ar), 7.97 (d, *J*=8.2 Hz, 1H, Ar), 8.04 (br s, 1H, Ar); ^13^C NMR (125 MHz, CD_3_OD): *δ* 15.7 (2 C), 17.1 (1 C), 36.4 (1 C), 48.4 (1 C), 51.4 (1 C), 126.0 (1 C), 126.5 (1 C), 126.8 (1 C), 127.3 (1 C), 127.4 (1 C), 127.71 (1 C), 127.75 (2 C), 128.6 (1 C), 128.9 (2 C), 129.3 (1 C), 133.3 (1 C), 133.4 (1 C), 133.6 (1 C), 135.3 (1 C), 169.6 (1 C); HRMS (QTOF, *m/z*) calcd for C_23_H_27_N_2_O: 347.2118 ([M+H]^+^); found 347.2116. Anal. Calcd for C_23_H_26_N_2_O⋅HCl: C, 72.14; H, 7.11; N, 7.32; Found: C, 71.83; H, 7.06; N, 7.13.

#### *tert*‐Butyl (4‐hydroxybutan‐2‐yl)carbamate (14)

3‐[(*tert*‐Butoxycarbonyl)amino]butyric acid (**6**) (1.2 g, 5.9 mmol) was added under N_2_ atmosphere to a stirred and cold suspension of LiAlH_4_ (0.449 g, 11.8 mmol) in dry THF (40 mL). The reaction mixture was stirred at 0 °C for 30 min and at room temperature for 15 h. Then it was quenched by the careful addition of cold water until the end of gas evolution. The residue was removed by filtration and the filtrate, diluted with H_2_O, was extracted several times with EtOAc. The combined organic layers were dried over Na_2_SO_4_ and concentrated under vacuum to give 0.996 g (89 %) of a yellowish oil. Spectrometric and spectroscopic data were in agreement with those reported in the literature.^[64]^

#### *tert*‐Butyl [4‐(2,6‐dimethylphenoxy)butan‐2‐yl]carbamate (15)

A solution of diisopropyl azodicarboxylate (DIAD, 0.69 g, 3.42 mmol) in dry THF (13 mL) was added dropwise to a solution of *tert*‐butyl (4‐hydroxybutan‐2‐yl)carbamate (**14**) (0.43 g, 2.28 mmol), 2,6‐dimethylphenol (0.41 g, 3.42 mmol), and triphenylphosphine (0.9 g, 3.42 mmol) in dry THF (22 mL) under N_2_ atmosphere at room temperature. The reaction mixture was stirred for 24 h and then concentrated in vacuo. Et_2_O was added to the residue and the solid filtered off. The filtrate was evaporated and the residue was purified by silica gel column chromatography (EtOAc/hexane, 1 : 9) to give 0.468 g of a white solid (70 %): mp 85–86 °C; ^1^H NMR (300 MHz): *δ* 1.24 (d, *J*=6.6 Hz, 3H, C*H*_3_CH), 1.44 (s, 9H, *t*‐Bu), 1.80–1.92 (m, 1H, C*H*HCH), 1.93–2.06 (m, 1H, CH*H*CH), 2.27 (s, 6H, C*H*_3_Ar), 3.74–3.81(m, 1H, C*H*), 3.82–3.98 (m, 2H, C*H*_2_O), 4.76 (br s, 1H, N*H*), 6.87–7.03 (m, 3H, Ar); ^13^C NMR (125 MHz): *δ* 16.3 (2 C), 21.4 (1 C), 28.4 (3 C), 37.1 (1 C), 44.7 (1 C), 69.3 (1 C), 79.0 (1 C), 123.8 (1 C), 128.8 (2 C), 130.9 (2 C), 155.4 (1 C), 155.8 (1 C); GC‐MS (70 eV) *m/z* (%) 219 (M^+^−74, <6), 116 (100).

#### 4‐(2,6‐Dimethylphenoxy)butan‐2‐amine (16)

##### Method A: Prepared as reported for 8 starting from 15. Yellowish oil (57 %)

Method B: To a stirred solution of **20** (0.83 g, 2.58 mmol) in MeOH (10 mL), glacial AcOH (0.3 mL, 5.16 mmol) and N_2_H_4_⋅H_2_O (10.3 mmol) were added and the mixture was kept under reflux for 4 h. The solid residue was filtered off. After evaporation of the filtrate, the residue was taken up with Et_2_O and extracted with 2 M HCl; then, the aqueous phase was treated with 2 M NaOH and extracted several times with Et_2_O. The combined organic layers were dried (Na_2_SO_4_) and concentrated under vacuum to give **16** (61 %) as a yellowish oil. Spectrometric and spectroscopic data were in agreement with those reported in the literature.^[17]^

#### *N*‐[(Naphthalen‐1‐yl)methyl]‐4‐[(2,6‐dimethyl)phenoxy]butan‐2‐amine (17 a)

Prepared as reported for **13 a** starting from **11** and **16**. Yellowish oil (23 %): ^1^H NMR (300 MHz): *δ* 1.31 (d, *J*=6.4 Hz, 3H, C*H*_3_CH), 1.85–1.95 (m, 2H, C*H*HCH+NH), 2.05–2.15 (m, 1H, CH*H*CH), 2.23 (s, 6H, C*H*_3_Ar), 3.19 (sextet, *J*=6.3 Hz, 1H, C*H*), 3.87 (apparent dt, 2H, C*H*_2_O), 4.23 (d, *J*=12.9 Hz, 1H, C*H*HNH), 4.37 (d, *J*=12.9 Hz, 1H, CH*H*NH), 6.86–7.02 (m, 3H, ArO), 7.38–7.52 (m, 4H, Ar), 7.77 (d, *J*=8.2 Hz, 1H, Ar), 7.82–7.88 (m, 1H, Ar), 8.12–8.18 (m, 1H, Ar); GC‐MS (70 eV) *m/z* (%) 333 (M^+^, 7), 141 (100). The corresponding hydrochloride (**17 a**⋅HCl) was obtained dissolving the free amine in 1 mL of 2 M HCl and then azeotropically removing water (abs EtOH/toluene) affording a solid which was recrystallized from abs EtOH/Et_2_O to give 0.23 g of white crystals (10 %) as white crystals: mp 183–184 °C; ^1^H NMR (500 MHz, CD_3_OD): *δ* 1.63 (d, *J*=6.4 Hz, 3H, C*H*_3_CH), 2.05–2.12 (m, 1H, C*H*HCH), 2.23 (s, 6H, C*H*_3_Ar), 2.42–2.54 (m, 1H, CH*H*CH), 3.82–3.90 (m, 1H, C*H*), 3.90–3.95 (m, 1H, C*H*HO), 3.96–4.02 (m, 1H, CH*H*O), 4.82 (d, *J*=12.4 Hz, AB system, 1H, C*H*HNH), 4.84 (d overlapping CD_3_OD, *J*=12.4 Hz, AB system, 1H, CH*H*NH), 6.90–7.02 (m, 3H, ArO), 7.55–7.62 (m, 3H, Ar), 7.73 (d, *J*=6.3 Hz, 1H, Ar), 7.95–8.00 (m, 1H, Ar), 8.01 (d, *J*=8.3 Hz, 1H, Ar), 8.12–8.18 (m, 1H, Ar); ^13^C NMR (125 MHz, CD_3_OD): *δ* 15.1 (2 C), 15.4 (1 C), 33.3 (1 C), 45.4 (1 C), 53.4 (1 C), 68.1 (1 C), 122.2 (1 C), 124.0 (1 C), 125.1 (1 C), 126.2 (1 C), 127.1 (1 C), 127.4 (1 C), 128.6 (1 C), 128.8 (2 C), 129.0 (1 C), 130.22 (2 C), 130.24 (1 C), 131.2 (1 C), 134.1 (1 C), 155.1 (1 C). HRMS (QTOF, *m/z*) calcd for C_23_H_28_NO: 334.2165 ([M+H]^+^); found 334.2191. Anal. Calcd for C_23_H_27_NO⋅HCl⋅1.5H_2_O: C, 69.59; H, 7.87; N, 3.53; Found: C, 69.30; H, 7.52; N, 3.17.

#### *N*‐[(Naphthalen‐2‐yl)methyl]‐4‐[(2,6‐dimethyl)phenoxy]butan‐2‐amine (17 b)

Prepared as reported for **13 a** starting from **12** and **16**. Yellowish oil (45 %): ^1^H NMR (300 MHz): *δ* 1.24 (d, *J*=6.3 Hz, 3H, C*H*_3_CH), 1.81 (s, 1H, NH), 1.85–1.95 (m, 1H, C*H*HCH), 2.00–2.10 (m, 1H, CH*H*CH), 2.26 (s, 6H, C*H*_3_Ar), 3.10 (sextet, *J*=6.3 Hz, 1H, C*H*), 3.88 (apparent dt, 2H, C*H*_2_O), 3.95 (d, *J*=12.0 Hz, 1H, C*H*HNH), 4.07 (d, *J*=12.0 Hz, 1H, CH*H*NH), 6.88–7.02 (m, 3H, ArO), 7.42–7.52 (m, 3H, Ar), 7.75–7.85 (4H, Ar); GC‐MS (70 eV) *m/z* (%) 333 (M^+^, 5), 141 (100). The corresponding hydrochloride (**17 b**⋅HCl), obtained as described for **16**⋅HCl, was recrystallized from abs EtOH/Et_2_O giving 0.135 g of white crystals (31 %): mp 178–179 °C; ^1^H NMR (300 MHz, CD_3_OD): δ 1.58 (d, *J*=6.6 Hz, 3H, C*H*_3_), 1.99–2.10 (m, 1H, C*H*HCH), 2.20 (s, 6H, C*H*_3_Ar), 2.44–2.54 (m, 1H, CH*H*CH), 3.64–3.78 (m, 1H, C*H*), 3.84–3.94 (m, 2H, C*H*_2_O), 4.44 (d, *J*=13.2 Hz, 1H, C*H*HNH), 4.50 (d, *J*=13.2 Hz, 1H, CH*H*NH), 6.82–7.00 (m, 3H, ArO), 7.48–7.58 (m, 2H, Ar), 7.65 (dd, *J*=8.5, 1.6 Hz, 1H, Ar), 7.88 (d, *J*=9.3 Hz, 1H, Ar), 7.90 (d, *J*=9.3 Hz, 1H, Ar), 7.95 (d, *J*=8.5 Hz, 1H, Ar), 8.08 (br s, 1H); ^13^C NMR (75 MHz, CD_3_OD): δ 19.3 (2 C), 19.5 (1 C), 37.4 (1 C), 52.4 (1 C), 56.4 (1 C), 72.0 (1 C), 128.1 (1 C), 130.5 (1 C), 130.7 (1 C), 131.0 (1 C), 131.6 (1 C), 131.9 (1 C), 132.7 (1 C), 132.8 (2 C), 133.0 (1 C), 133.7 (1 C), 134.5 (2 C), 137.5 (1 C), 137.7 (1 C), 159.4 (1 C). HRMS (QTOF, *m/z*) calcd for C_23_H_28_NO: 334.2165 ([M+H]^+^); found 334.2173. Anal. Calcd for C_23_H_27_NO⋅HCl⋅0.25 H_2_O: C, 73.78; H, 7.67; N, 3.74; Found: C, 74.10; H, 7.51; N, 3.92.

#### 4‐(2,6‐Dimethylphenoxy)butan‐2‐ol (19)

To a stirred solution of 1,3‐butanediol (**18**) (1.0 g, 11.1 mmol), 2,6‐dimethylphenol (2.03 g, 16.7 mmol) and triphenylphosphine (4.36 g, 16.7 mmol) in dry THF (40 ml), under N_2_ atmosphere, a solution of DIAD (3.3 mL, 16.7 mmol) in dry THF (20 ml) was added dropwise. The mixture was stirred at room temperature for 24 h. The solvent was then evaporated under reduced pressure, ether was added and the precipitate formed was filtered off. The filtrate was evaporated in vacuo and the residue was purified by silica gel column chromatography (eluent Et_2_O/hexane 3 : 7) to give 1.23 g of a colorless oil (57 %): ^1^H NMR (500 MHz): δ 1.30 (d, *J*=6.4 Hz, 3H, C*H*_3_), 1.86–1.92 (m, 1H, C*H*HCH), 1.93–2.02 (m, 1H, CH*H*CH), 2.29 (s, 6H, C*H*_3_Ar), 2.72 (br s, exch with D_2_O, 1H, O*H*), 3.90–4.00 (m, 2H, C*H*_2_O), 4.15–4.25 (m, 1H, C*H*), 6.85–7.02 (m, 3H, Ar); ^13^C NMR (125 MHz): δ 16.2 (2 C), 23.7 (1 C), 38.9 (1 C), 67.2 (1 C), 70.7 (1 C), 124.0 (1 C), 128.9 (2 C), 130.8 (2 C), 155.5 (1 C); GC‐MS (70 eV) *m/z* (%) 194 (M^+^, 10), 122 (100).

#### 2‐[4‐(2,6‐Dimethylphenoxy)butan‐2‐yl]‐1*H*‐isoindole‐1,3(2*H*)‐dione (20)

To a stirred solution of 4‐(2,6‐dimethylphenoxy)butan‐2‐ol (**19**) (1.12 g, 5.77 mmol), phthalimide (1.27 g, 8.65 mmol), and triphenylphosphine (2.27 g, 8.65 mmol) in dry THF (40 ml), under N_2_ atmosphere, a solution of DIAD (1.7 mL, 8.65 mmol) in dry THF (20 ml) was added dropwise. The mixture was stirred at room temperature for 24 h. The solvent was then evaporated under reduced pressure, ether was added and the precipitate formed was filtered off. The filtrate was evaporated in vacuo and the residue was purified by silica gel column chromatography (eluent Et_2_O/hexane 1 : 9, then 2 : 8) to give 0.86 g of a colorless oil (46 %): ^1^H NMR (500 MHz): δ 1.55 (d, *J*=6.8 Hz, 3H, C*H*_3_), 2.19 (s, 6H, C*H*_3_Ar), 2.22–2.32 (m, 1H, C*H*HCH), 2.65–2.72 (m, 1H, CH*H*CH), 3.76 (t, *J*=6.4 Hz, 2H, C*H*_2_O), 4.68–4.75 (m, 1H, C*H*), 6.82–6.88 (m, 3H, ArO), 7.66–7.72 (m, 2H, Ar), 7.80–7.84 (m, 2H, Ar); ^13^C NMR (125 MHz): δ 16.2 (2 C), 19.0 (1 C), 34.1 (1 C), 44.8 (1 C), 69.4 (1 C), 123.1 (2 C), 123.7 (1 C), 128.7 (2 C), 130.8 (2 C), 132.0 (2 C), 133.9 (2 C), 156.0 (1 C), 168.5 (2 C); GC‐MS (70 eV) *m/z* (%) 323 (M^+^, <1), 202 (100).

### Biology

**Patch clamp experiments**. Exploratory compounds were tested on sodium currents recorded in HEK293T cells (human embryonic kidney cell line) permanently expressing the wild‐type human Nav1.4 sodium channel, as previously described.[^14^, ^61^] Whole‐cell sodium currents were recorded at room temperature (∼22 °C) using Axon hardware and software (Axon Instruments Inc., Union City, CA, USA). Bath solution contained (mM): 150 NaCl, 4 KCl, 2 CaCl_2_, 1 MgCl_2_, 5 HEPES and 5 glucose (pH 7.4). Pipette solution contained (mM): 120 CsF, 10 CsCl, 10 NaCl, 5 EGTA, and 5 HEPES (pH 7.2). Pipette resistance was 2–4 mΩ. Stabilization of the whole‐cell configuration was allowed for 5 minutes before to record sodium currents elicited from a holding potential of −120 mV using 25 ms‐long test pulses at −30 mV applied every 10 or 0.1 sec (0.1 and 10 Hz frequencies). The patched cell was continuously perfused by control or drug‐supplemented bath solution. To limit errors due to rundown and voltage‐dependence shifts, no more than two drug concentrations were tested on each cell. The *I*_DRUG_/*I*_CTRL_ ratio (mean±S.E.M. from at least 3 cells) was plotted against drug concentration. The relationships were fitted to a first‐order binding function

$$I{}_{\mathrm{drug}}\;/\;I{}_{\mathrm{ctrl}}=1/\left\{1+\left(\left[\mathrm{drug}\right]/\mathrm{IC}{}_{50}\right){}^{\mathrm{nH}}\right\}$$

The half‐maximum inhibitory concentration (IC_50_) and slope factor (nH) are given with the standard error of the fit.

**Permeability experiments**. Details for permeability experiments. Preparation of Caco‐2 monolayer. This procedure has been previously reported by Colabufo et al.^[65]^ Briefly, Caco‐2 cells were harvested with trypsin‐EDTA and seeded onto MultiScreen Caco‐2 assay system at a density of 10,000 cells per well. The culture medium was replaced every 48 h for the first 6 days and every 24 h thereafter. After 21 days in culture, the Caco‐2 monolayer was utilized for the permeability experiments. The Trans Epithelial Electrical Resistance (TEER) of the monolayers was measured daily before and after the experiment using an epithelial voltohmmeter (Millicell®‐ERS; Millipore, Billerica, MA). Generally, TEER values obtained are greater than 1000 Ω for a 21‐day culture. Drug transport experiment. Apical to basolateral (AB) and basolateral to apical (BA) permeability (Papp) of drugs were measured at 120 min and at various drug concentrations (1–100 μM). Drugs were dissolved in Hanks’ balanced salt solution (HBSS, pH 7.4) and sterile filtered. After 21 days of cell growth, the medium was removed from filter wells and from the receiver plate. The wells were filled with 75 μL of fresh HBSS buffer and the receiver plate with 250 μL per well of the same buffer. This procedure was repeated twice, and the plates were incubated at 37 °C for 30 min. After incubation, the HBSS buffer was removed and drug solutions added to the filter well (75 μL). HBSS without the drug was added to the receiver plate (250 μL). The plates were incubated at 37 °C for 120 min. After incubation, samples were removed from the apical (filter well) and basolateral (receiver plate) sides of the monolayer and stored at −20 °C up to analysis. Compounds were lyophilized at −52 °C and 0.06 bars for 24 h and desalted by adding 500 μL of CH_3_CN (in order to remove phosphate salts coming from HBSS), then 500 μL of Ammonium Acetate (50 mM, pH 5) were added. The resulting solutions were analyzed by ESI‐MS in order to determine the concentration of drug in the samples. The concentration of tested compounds was measured using UV spectroscopy. The apparent permeability (*P*_app_), in units of nm per second, was calculated using the following equation:

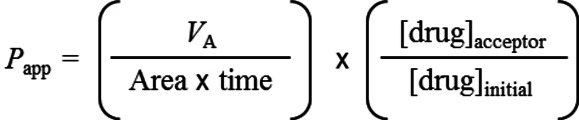




where *V*_A_ is the volume (in mL) in the acceptor well; Area is the surface area of the membrane (0.11 cm^2^ of the well); time is the total transport time in seconds (7200 s); [drug]_acceptor_ is the concentration of the drug measured by ESI‐MS analyses or UV spectroscopy; [drug]_initial_ is the initial drug concentration (1 ⋅ 10^−4^ M) in the apical or basolateral wells.

**Cell culture**. Cervical cancer cell lines (HeLa) were cultured in Dulbecco's modified eagle medium (DMEM) supplemented with 10 % fetal bovine serum (FBS), 1 % glutamine, and 1 % penicillin/streptomycin (all purchased from Euroclone, Italy) in a humidified incubator at 37 °C and 5 % CO_2_ and 95 % relative humidity.

**Flow cytometry analysis**. In order to detect ROS production after drugs treatment,[^66^, ^67^] cells at 3×10^5^ cells were seeded in 6‐well plates in DMEM with 10 % FBS and cultured overnight. The following day, cells were washed twice with PBS and kept in DMEM without FBS for 2 h. Then, cells were incubated with 1 μM and 50 μM concentrations of **13 a** and **17 a** in culture medium for short (6 h) and long (24 h) term. Antibiotics were omitted during pharmacological compound assays. After incubation period, cells were washed twice with HBSS, and stained with 5 μM of 2′,7′‐dichorofluorescein (DCFA‐DA) and dihydrorhodamine (DHR) (Thermo Fischer Scientific, Italy) for 30 min in dark. After washing, cells were resuspended in HBSS and analyzed using an Attune Acoustic Focusing Cytometer (Thermo Fisher Scientific) equipped with a 488 nm laser. Thereafter, DCFA‐DA stained cells were co‐stained with 1 μg/mL of 7‐aminoactinomycin D (7‐AAD) (Thermo Fischer Scientific, Italy), incubated on ice for 45 min in dark in order to asses viable and non‐viable cells, and then flow cytometry analysis was carried out.

### Molecular modelling

**Quantum mechanical calculations**. Calculations were performed according to previously developed procedures.[^51^, ^52^] Briefly, the model of compound **13 a** was built through the atomic fragments incorporated into Spartan’20 (Wavefunction Inc., Irvine, CA, USA) inner fragment library and assuming the suggested default starting geometries. The structure was optimized by molecular mechanics MMFF force field^[68]^ and then submitted to a Monte Carlo conformational distribution analysis using the default step sizes. All conformers in a window of 10 Kcal/mol above the global minimum conformation were retained. Redundant conformers^[52]^ were eliminated. The ab initio gas‐phase energy content of each survived conformation was then calculated at the RHF/6‐31G* level. Only conformers falling within a window of 5 kcal/mol above the absolute minimum were retained and submitted to RHF/6‐31G* geometry optimization. After removal of redundant conformers,^[52]^ the so‐obtained set of conformers underwent geometry optimization by density functional theory (DFT) implemented in Spartan’20 with B3LYP functional^[69]^ and the 6–31G* basis set^[70]^ in the gas phase. The optimized structures of representative **13 a** conformers were confirmed as real minima by IR frequency calculation (DFT EDF2/6‐31G*//DFT B3LYP/6‐31G*). The above geometry optimization was performed also applying the conductor‐like polarizable continuum model (C‐PCM; Spartan’20) to allow for polar solvating effect consideration in MeOH.[^71^, ^72^] ^1^H NMR spectra for the whole set of conformers were calculated in the gaseous phase DFT EDF2/6‐31G*//DFT B3LYP/6‐31G*). Relevant chemical shift values are reported in Table S1.

**Molecular modelling methods**. Compound **13 a**, with standard values of bond lengths and valence angles, were built, assign the (*R*) configuration to the carbon stereo centre, within Maestro software package^[73]^ as protonated skeleton and passed to Open Babel^[74]^ for a 10000 steps of Steepest Descent minimization using the Universal Force Field. Chain A of hNav1.4 (PDB code 6AGF) ion channel structure was passed to the Protein Preparation Wizard interface of Maestro, where water molecules were removed, and hydrogen atoms added, optimizing their position, and determining the protonation states of residues according to PROPKA prediction at pH 7.0. AMBER UNITED force field electrostatic charges^[75]^ were loaded on the protein structure, while the *molcharge* complement of QUACPAC^[76]^ was used in order to achieve Marsili‐Gasteiger charges for the inhibitors. Affinity maps were first calculated on a 0.375 Å spaced 50×50×50 Å^3^ cubic box, set on the Phe1586 barycentre, and the accessibility of the binding site was exploited throughout 1000 runs of Lamarckian Genetic Algorithm (LGA) implemented in AUTODOCK 4.2.6^[77]^ using the GPU‐OpenCL algorithm version.^[78]^ Explicit water contribution was taken into account according to the hydration force field,^[79]^ and the population size and the number of energy evaluations figures were set to 300 and 10000000, respectively. Among all the plausible ones, the best free energy of pose, according to the AUTODOCK scoring functions, was selected as representative of ligand binding mode, and the matching of this conformation with the lowest QM minima was obtained means of the shape matching algorithm ROCS.^[80]^

## Conflict of interest

The authors declare no conflict of interest.

## Supporting information

As a service to our authors and readers, this journal provides supporting information supplied by the authors. Such materials are peer reviewed and may be re‐organized for online delivery, but are not copy‐edited or typeset. Technical support issues arising from supporting information (other than missing files) should be addressed to the authors.

Supporting Information
