# Supplementary material for: Bioisosteric Modification of To042: Synthesis and Evaluation of Promising Use‐Dependent Inhibitors of Voltage‐Gated Sodium Channels
Source: ChemMedChem. 2021 Oct 5;16(23):3588–99. doi: 10.1002/cmdc.202100496 (PMC9293070; doi:10.1002/cmdc.202100496)
Supplement: Supplementary file 1 — Supporting Information [file CMDC-16-3588-s001.pdf]

ChemMedChem

Supporting Information

Bioisosteric Modification of To042: Synthesis and Evaluation of Promising Use-Dependent Inhibitors of Voltage-Gated Sodium Channels

Gualtiero Milani, Maria Maddalena Cavalluzzi,* Concetta Altamura, Antonella Santoro, Mariagrazia Perrone, Marilena Muraglia, Nicola Antonio Colabufo, Filomena Corbo, Elisabetta Casalino, Carlo Franchini, Isabella Pisano, Jean-François Desaphy, Antonio Carrieri, Alessia Carocci[+], and Giovanni Lentini[+]

Supporting Information

<u>Table of Contents</u>

^{1}H NMR, ^{13}C NMR, and GC-MS spectra of target compounds S2–S25

Table S1: ^{1}H NMR signal chemical shifts (ppm) in the aliphatic region of the **13a** spectrum S26

Figure S1: Flow cytometry analysis of ROS production by DCF-DA staining S27

Figure S2: Flow cytometry analysis of ROS production by DHR staining S28

Figure S3: Flow cytometry analysis of cell viability ROS by DCF-DA and 7-AAD co-staining S29

References S30

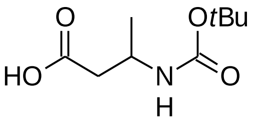

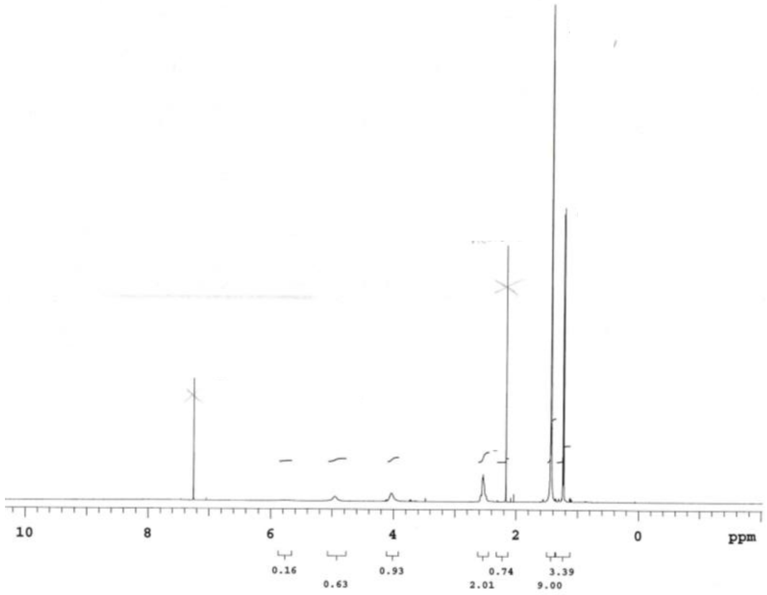

^{1}H NMR of **6** (500 MHz, $CDCl_3$)

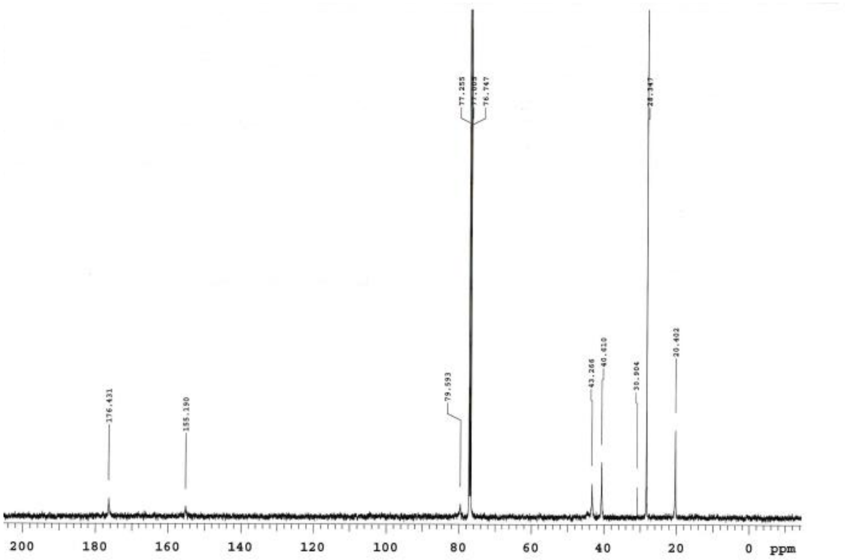

^{13}C NMR of **6** (125 MHz, $CDCl_3$)

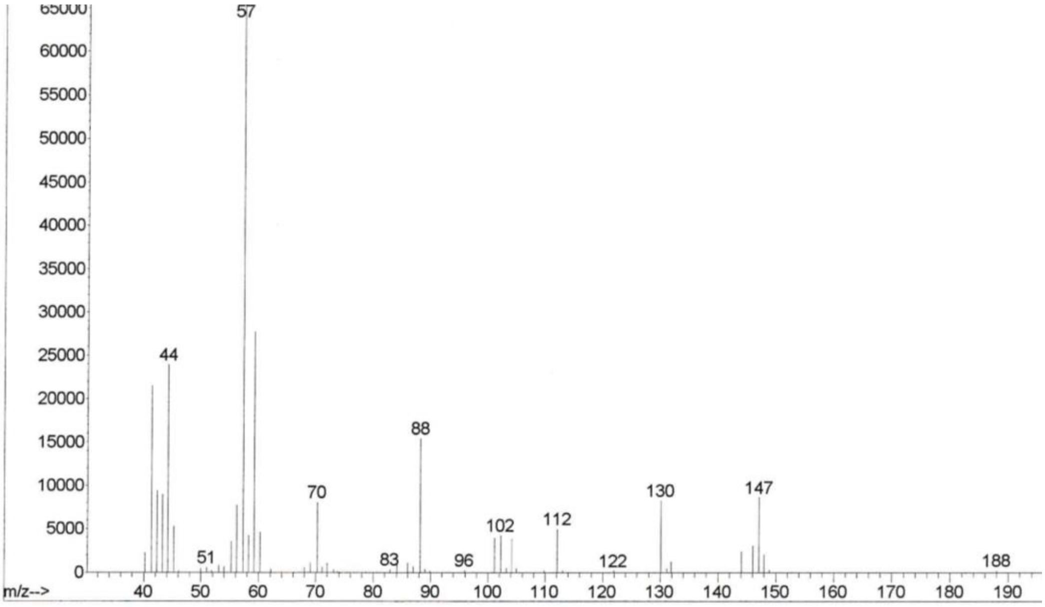

GC-MS (70 eV) of **6**

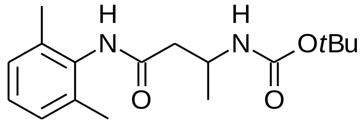

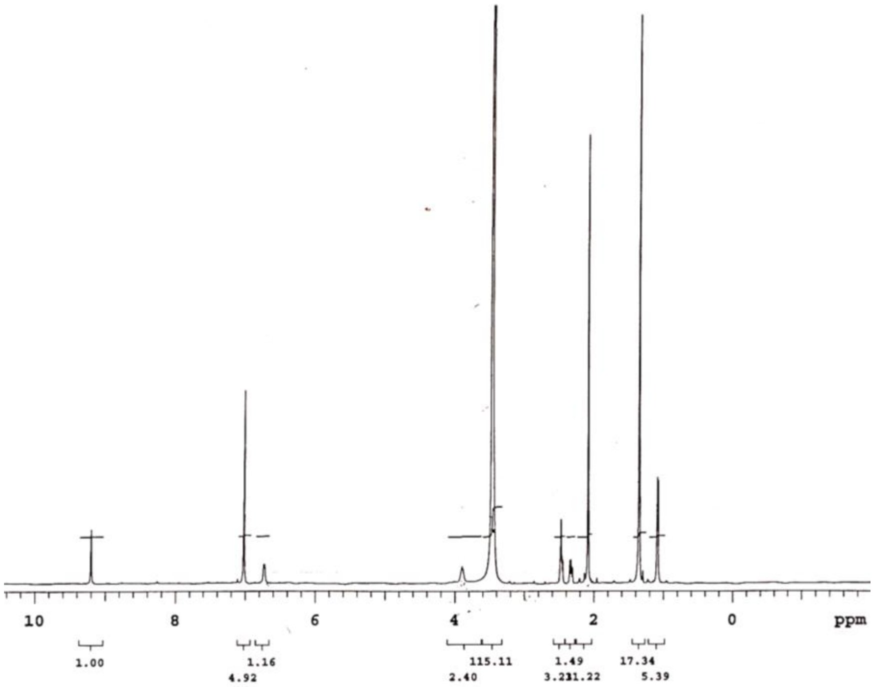

^{1}H NMR of **7** (500 MHz, DMSO-d_6)

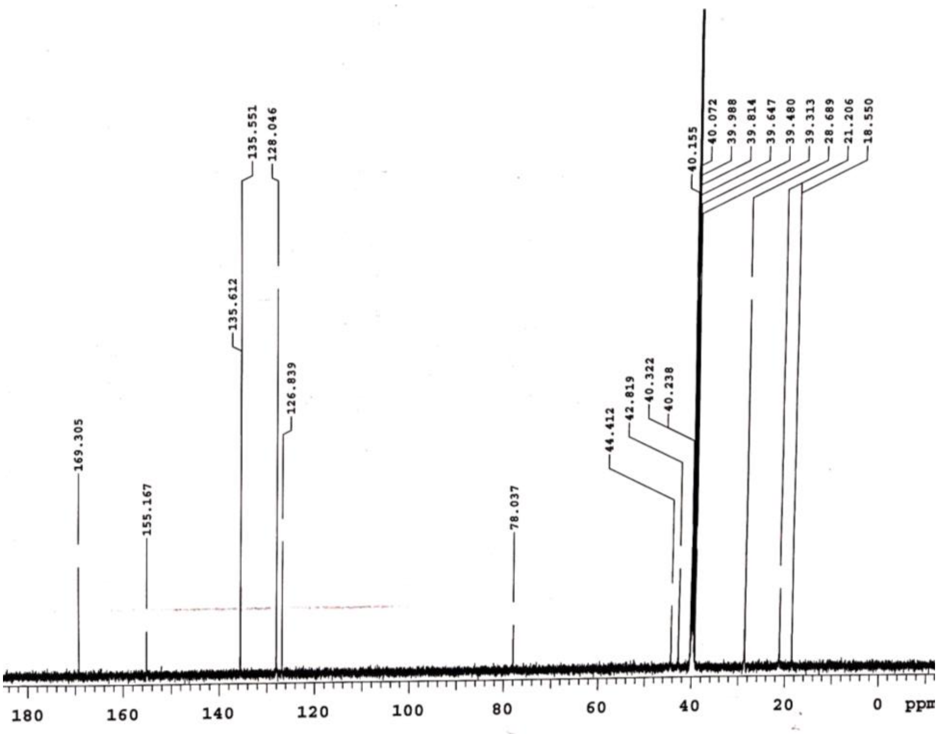

^{13}C NMR of **7** (125 MHz, DMSO-d_6)

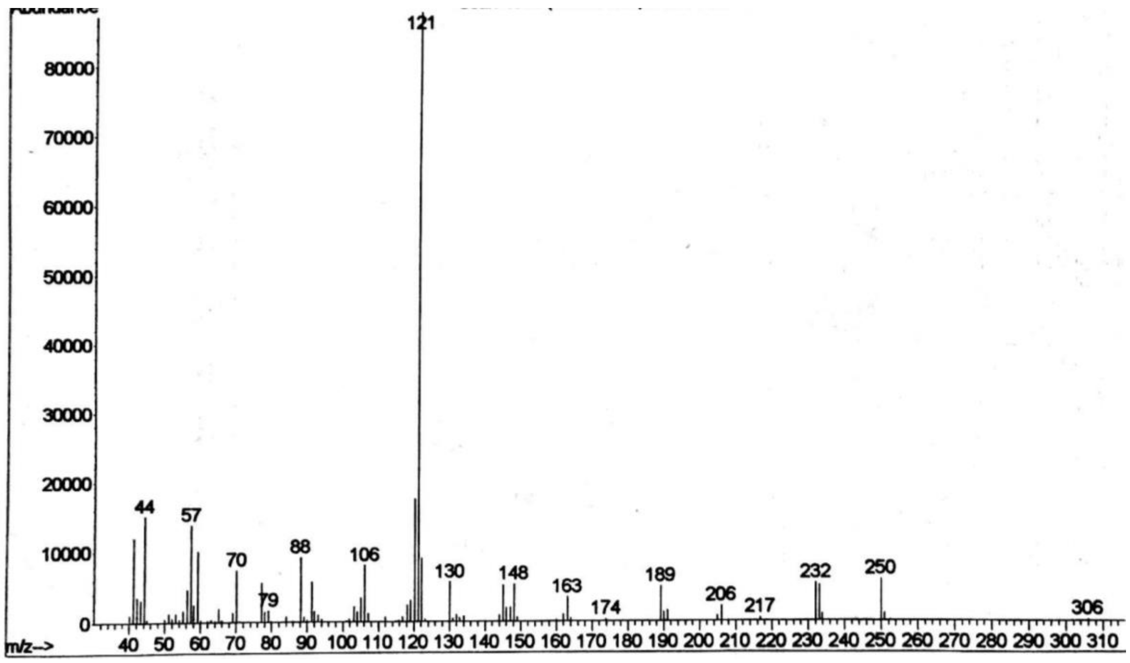

GC-MS (70 eV) of **7**

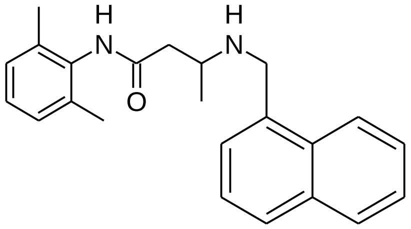

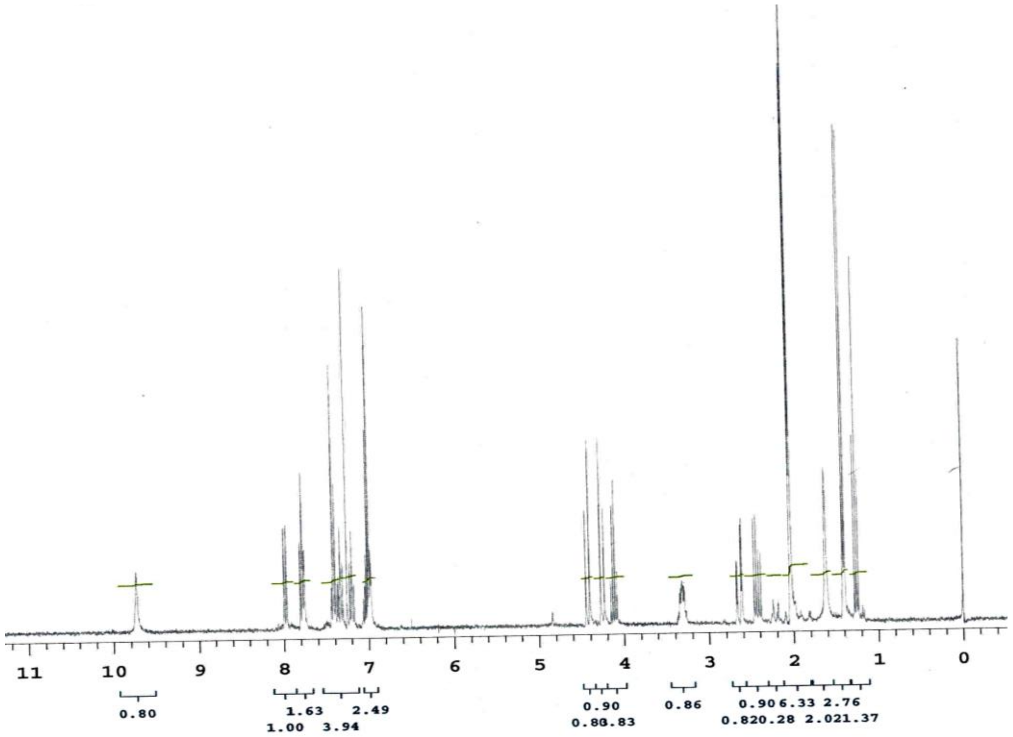

^{1}H NMR of **13a** (300 MHz, $CDCl_3$)

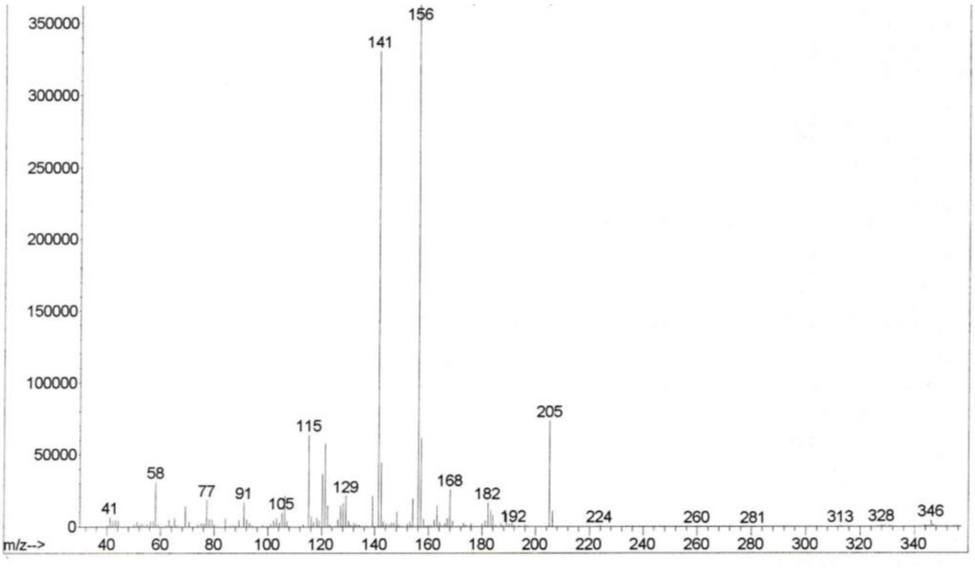

GC-MS (70 eV) of **13a**

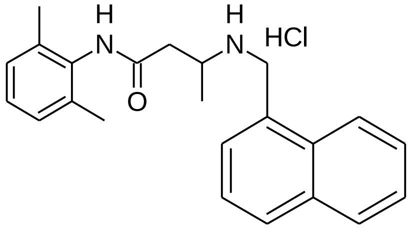

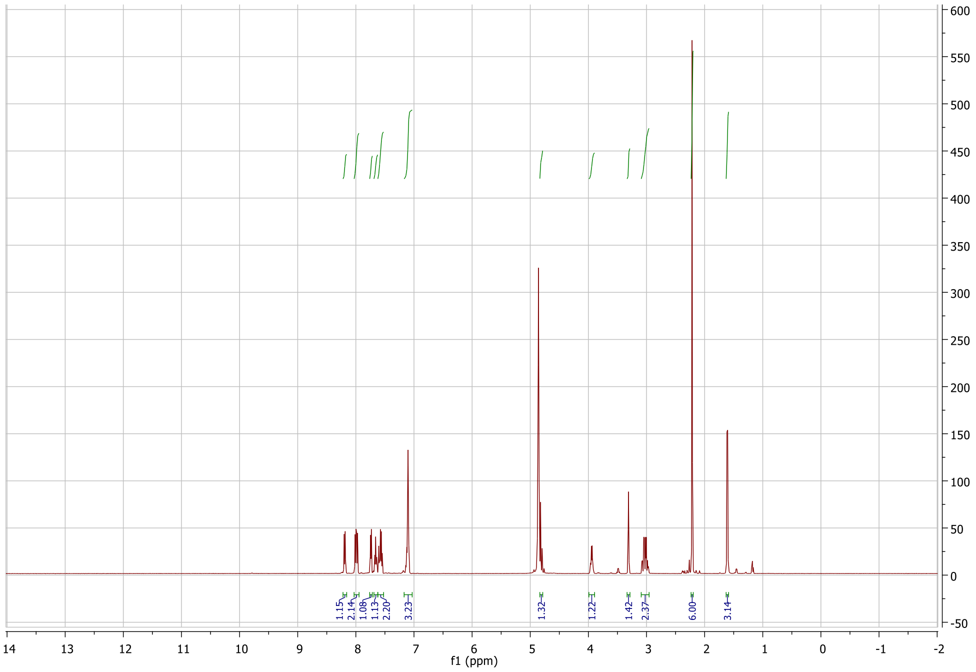

^{1}H NMR of **13a**·HCl (500 MHz, CD_3OD)

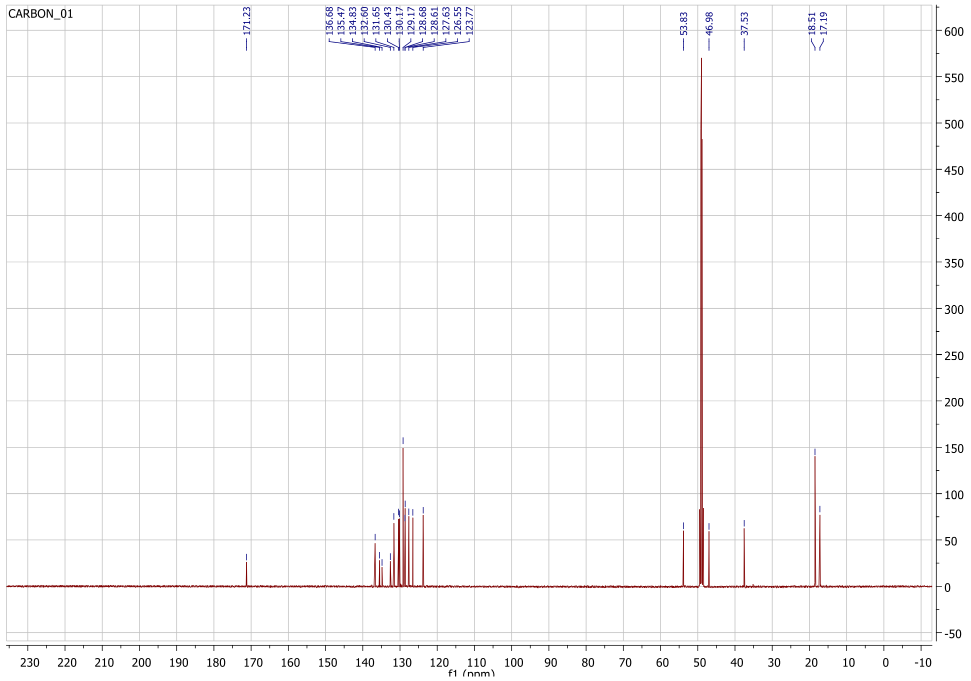

^{13}C NMR of **13a**·HCl (125 MHz, CD_3OD)

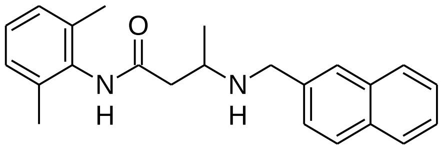

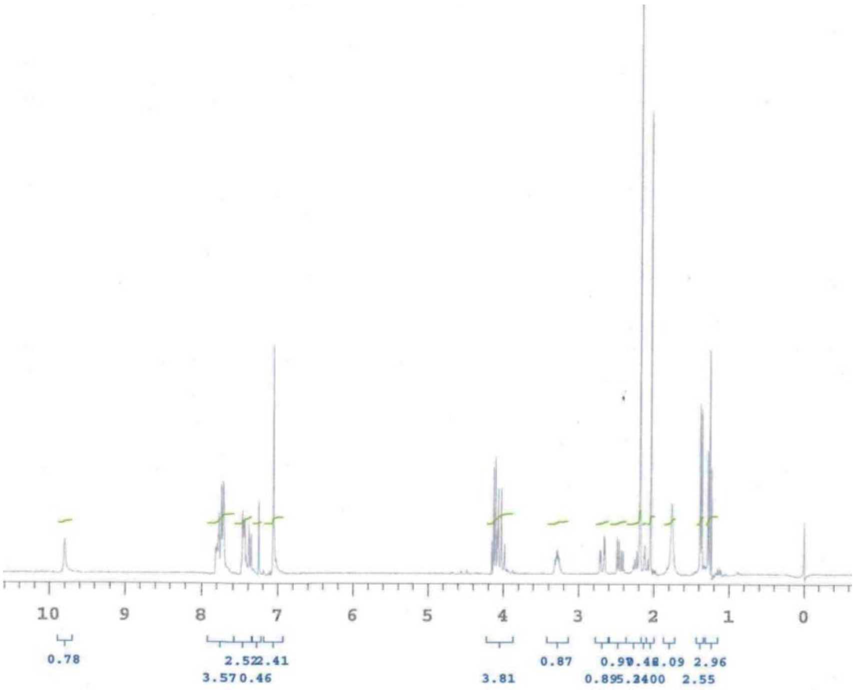

^{1}H NMR of **13b** (300 MHz, $CDCl_3$)

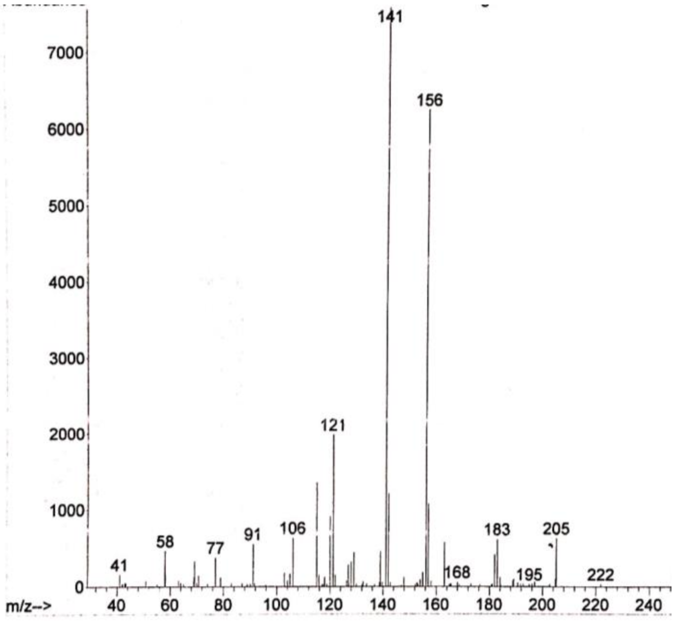

GC-MS (70 eV) of **13b**

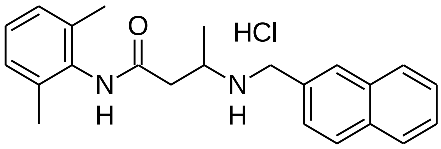

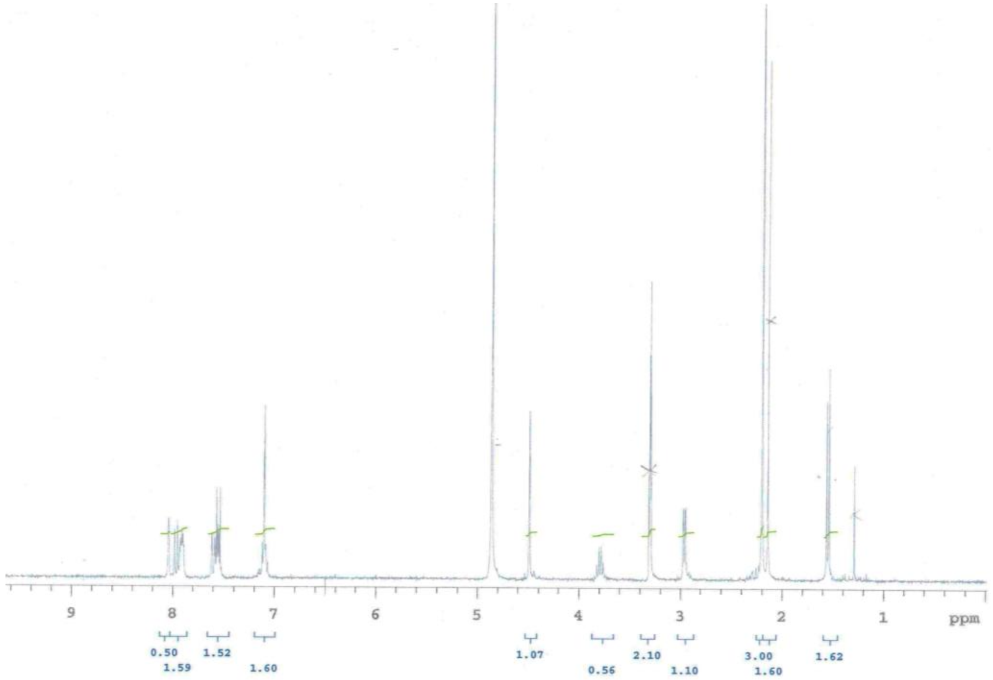

^{1}H NMR of **13b**·HCl (300 MHz, CD_3OD)

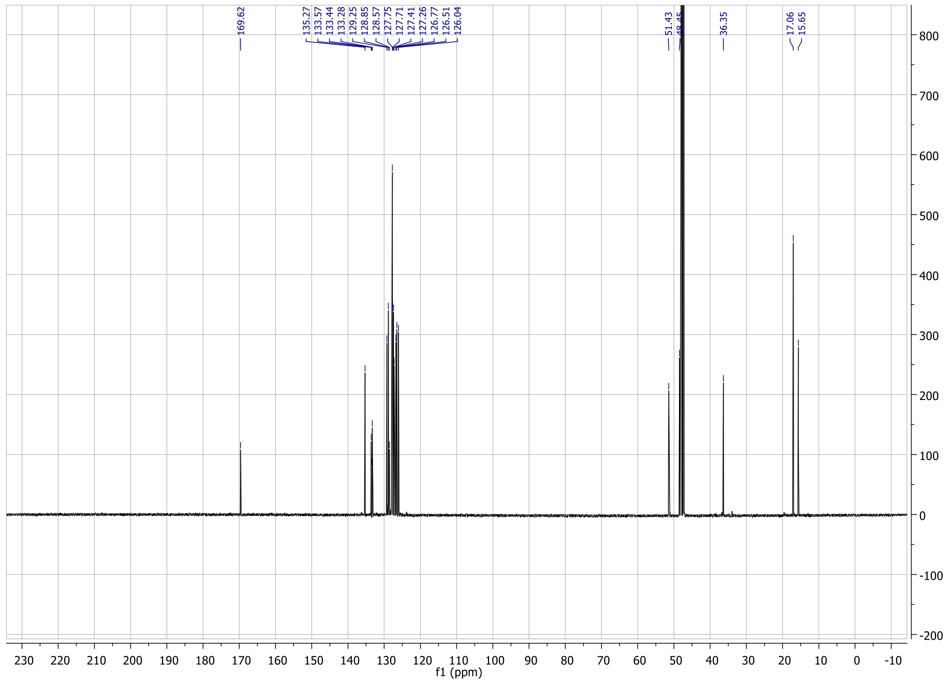

^{13}C NMR of **13b**·HCl (125 MHz, CD_3OD)

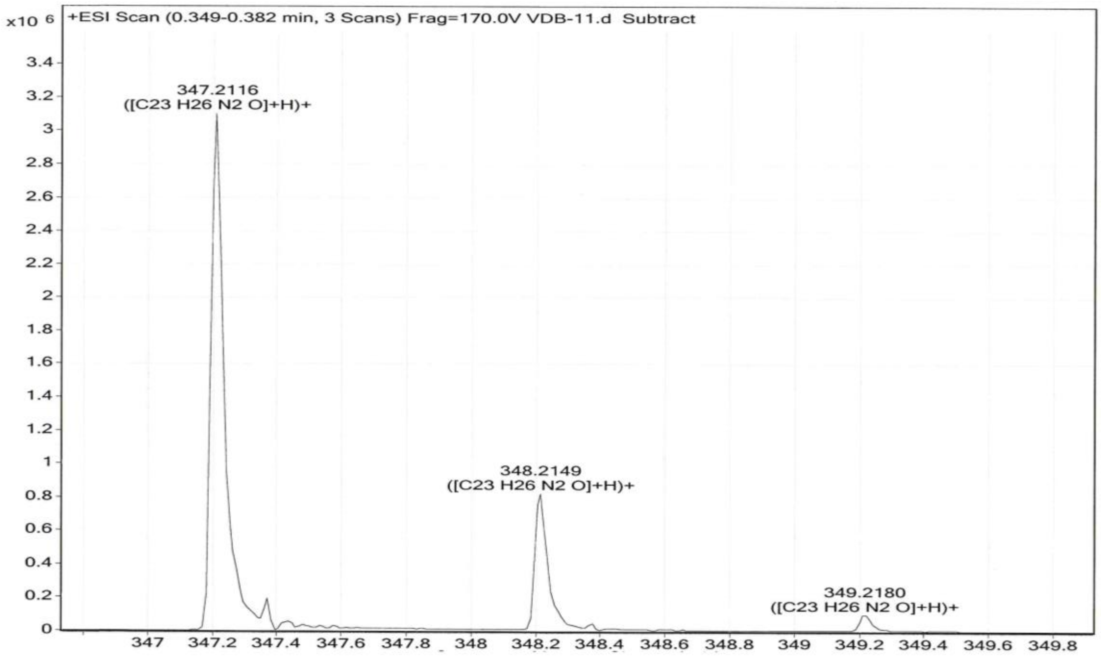

HRMS (QTOF) of **13b**·HCl

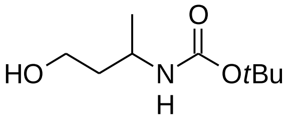

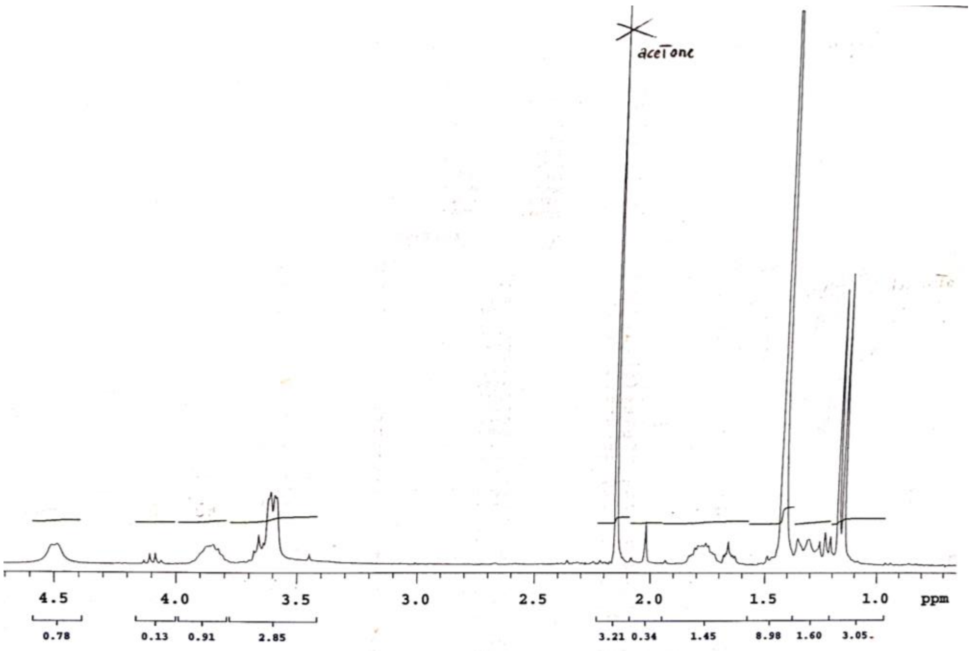

^{1}H NMR of **14** (300 MHz, $CDCl_3$)

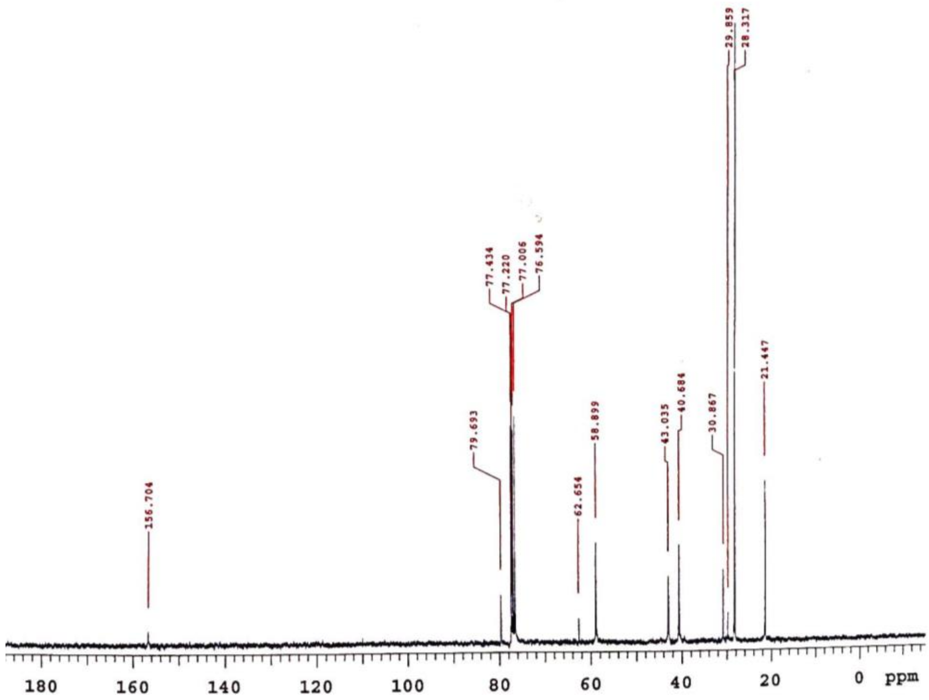

^{13}C NMR of **14** (75 MHz, $CDCl_3$)

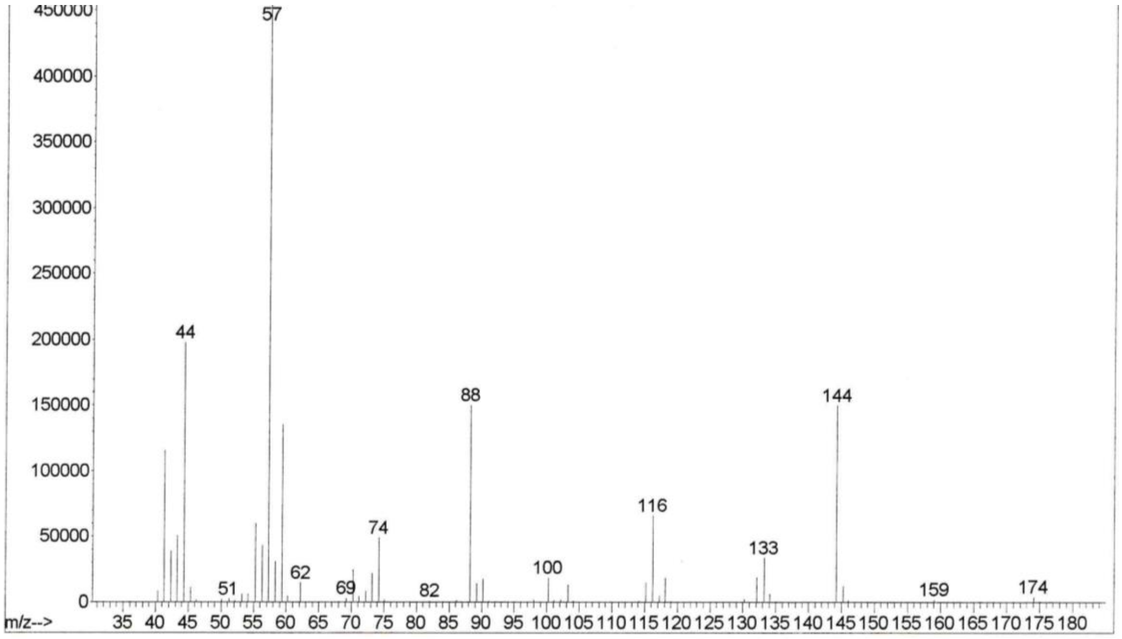

GC-MS (70 eV) of **14**

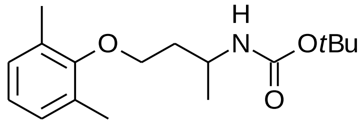

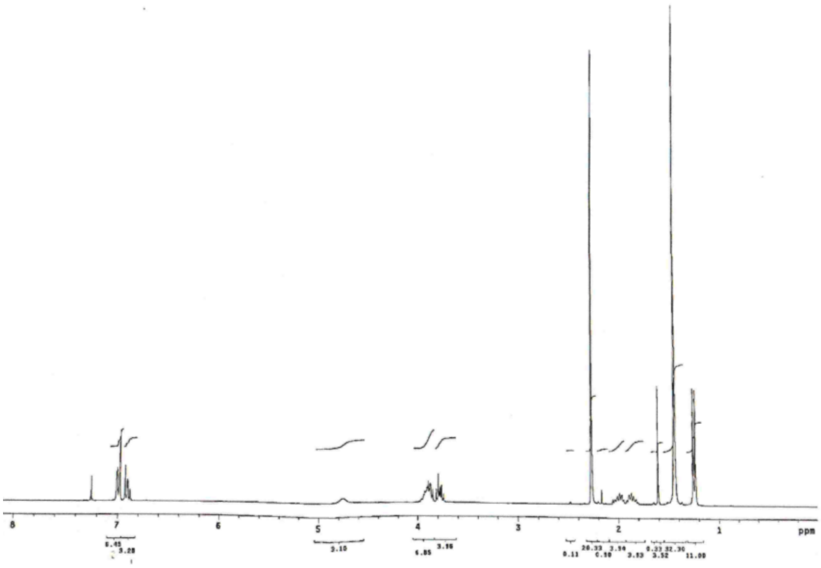

^{1}H NMR of **15** (300 MHz, $CDCl_3$)

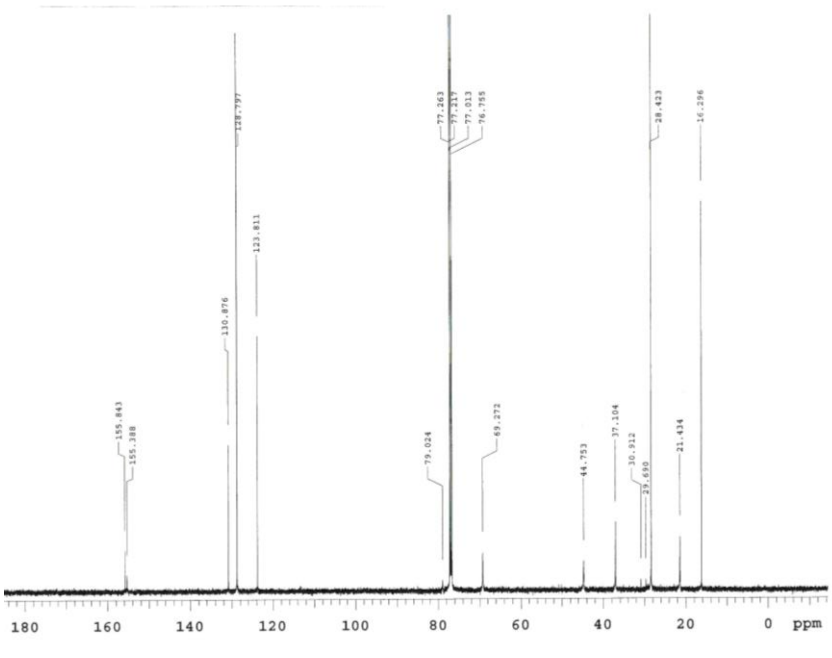

^{13}C NMR of **15** (125 MHz, $CDCl_3$)

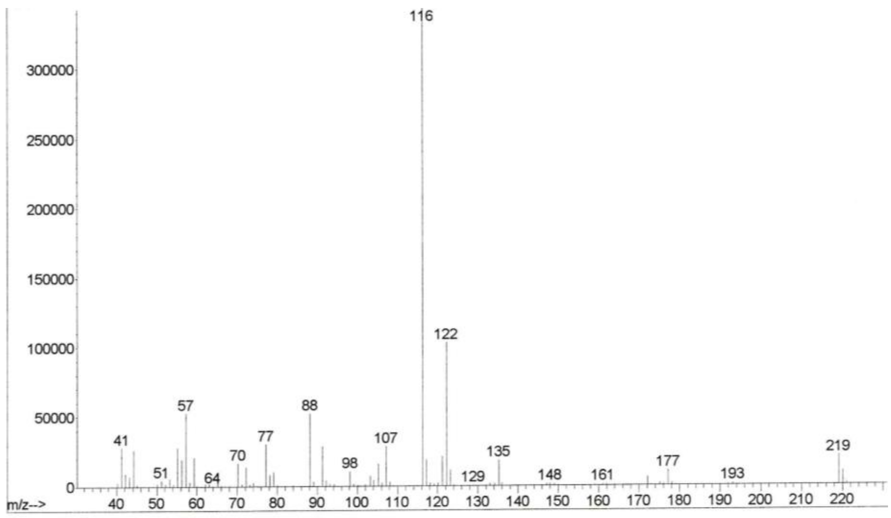

GC-MS (70 eV) of **15**

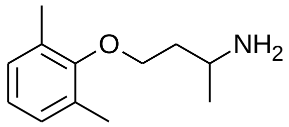

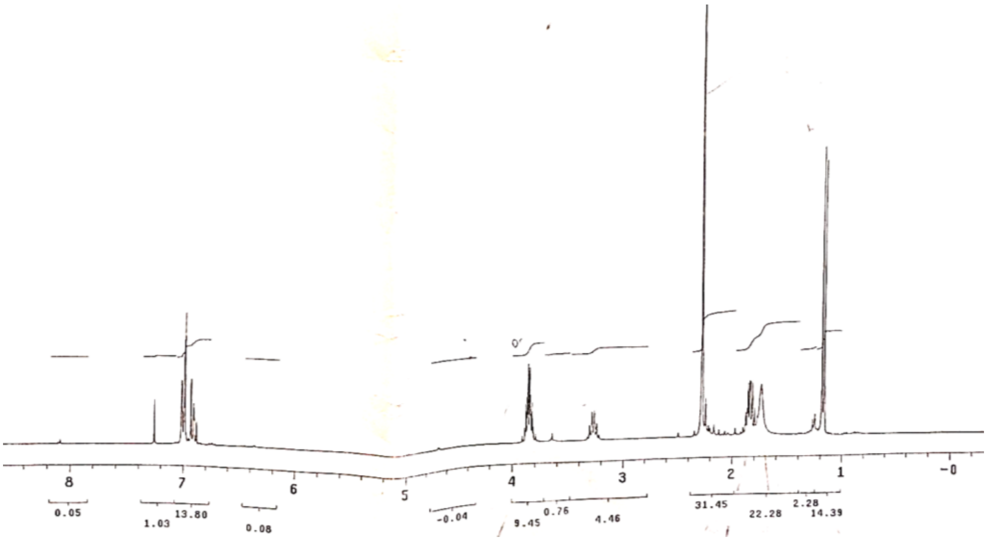

^{1}H NMR of **16** (300 MHz, $CDCl_3$)

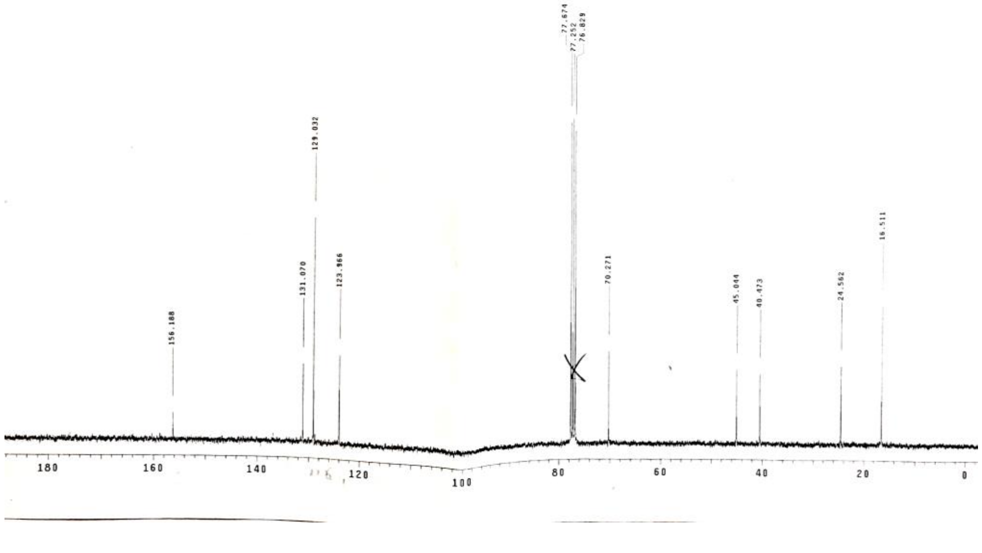

^{13}C NMR of **16** (75 MHz, $CDCl_3$)

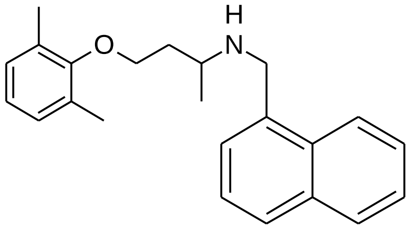

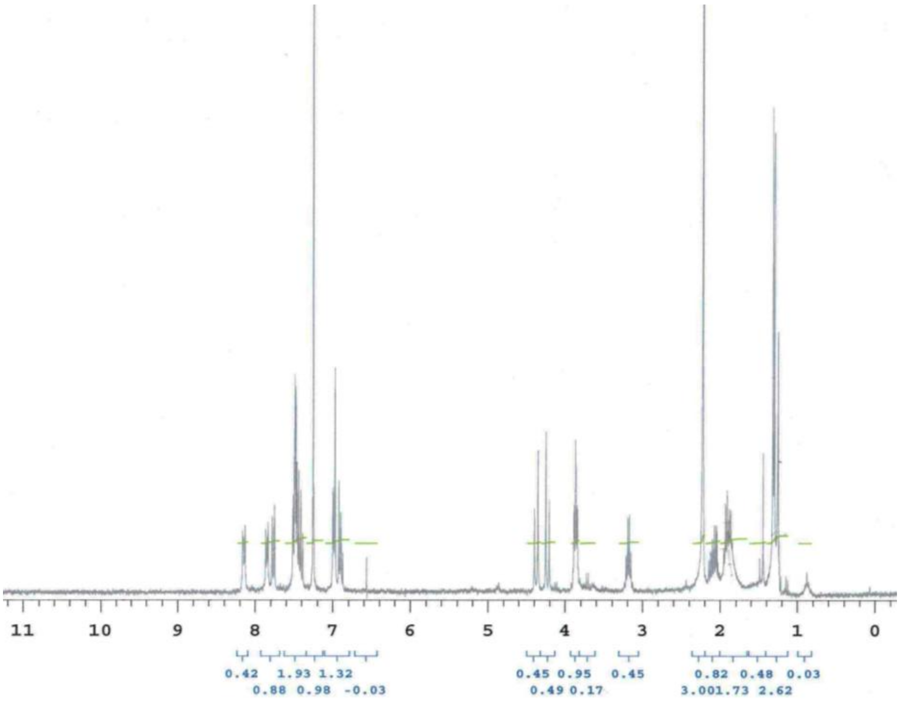

^{1}H NMR of **17a** (300 MHz, $CDCl_3$)

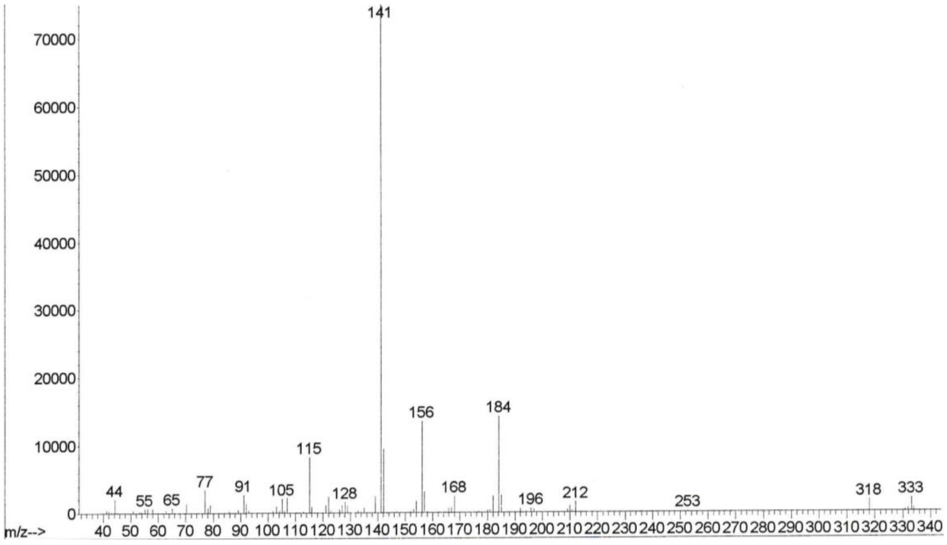

GC-MS (70 eV) of **17a**

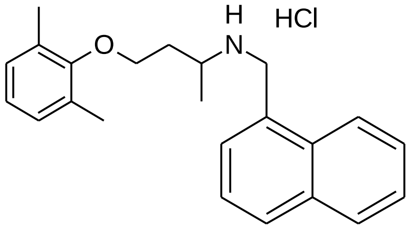

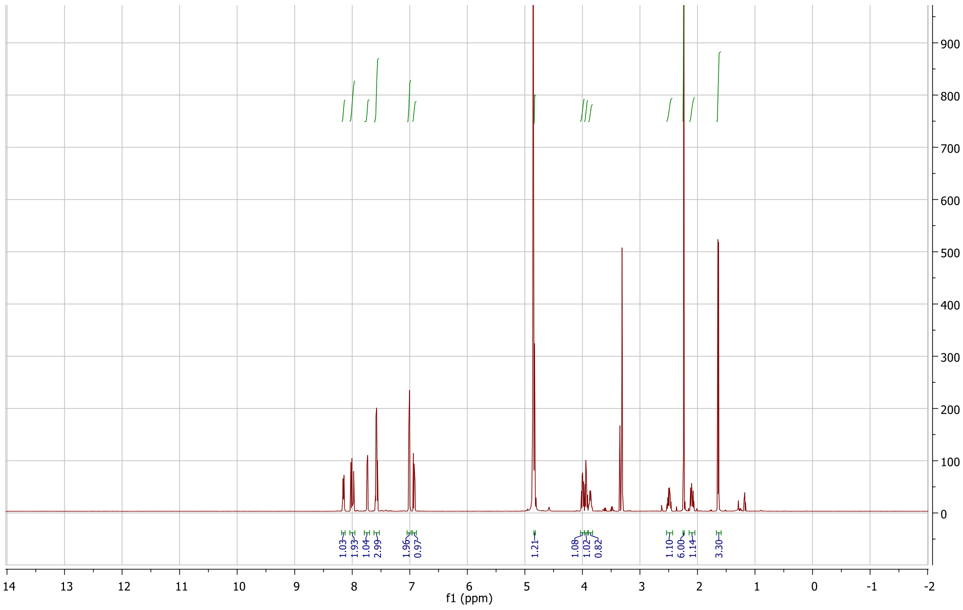

^{1}H NMR of **17a**·HCl (500 MHz, CD_3OD)

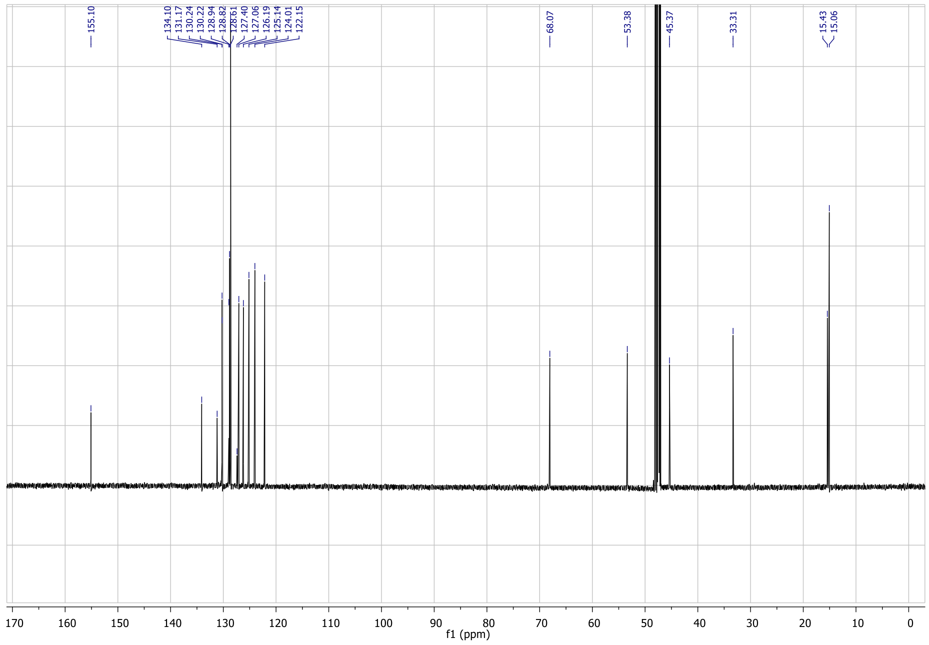

^{13}C NMR of **17a**·HCl (125 MHz, CD_3OD)

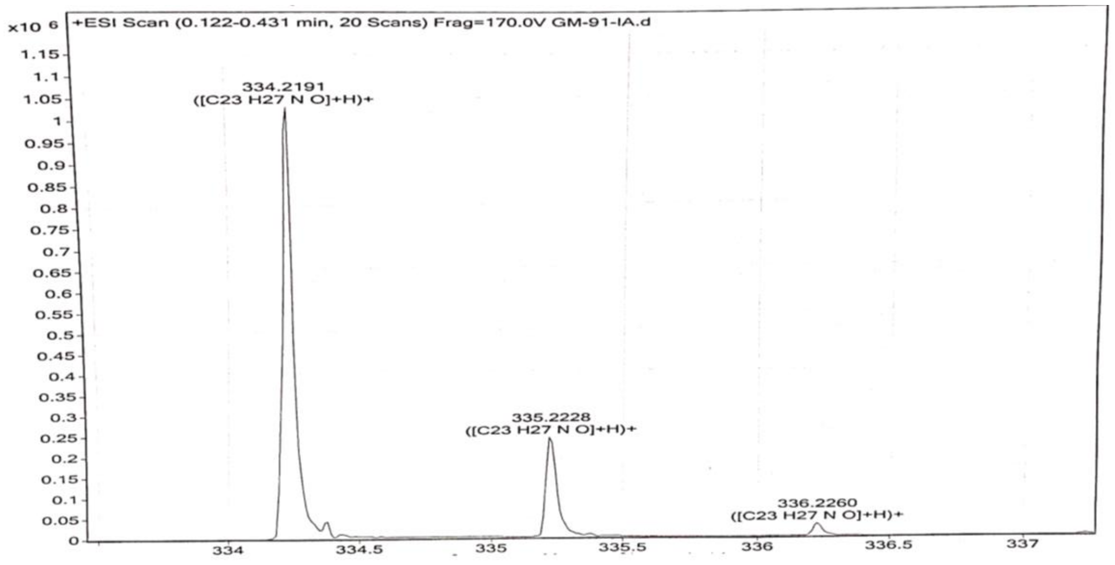

HRMS (QTOF) of compound **17a**·HCl

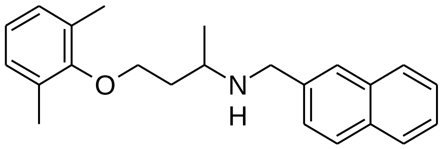

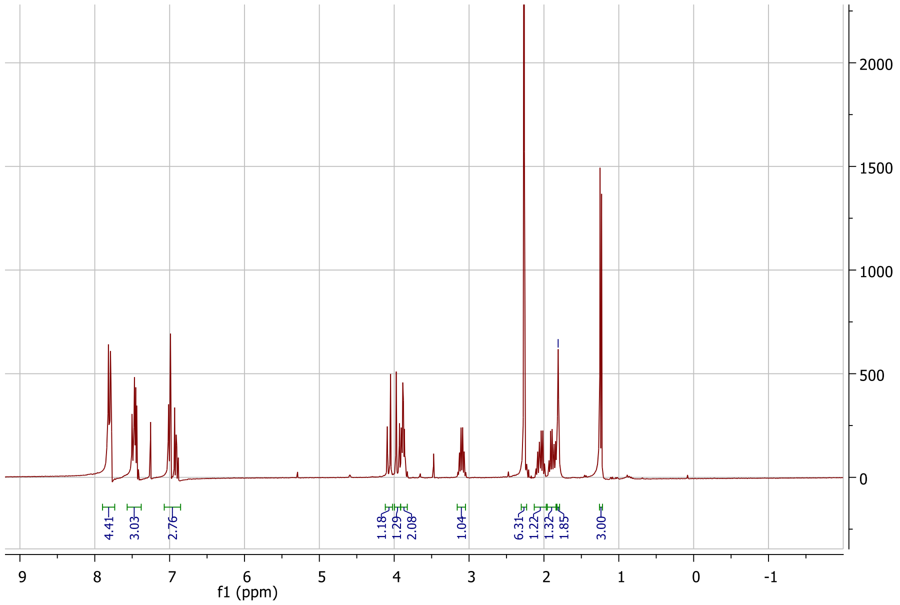

^{1}H NMR of **17b** (300 MHz, $CDCl_3$)

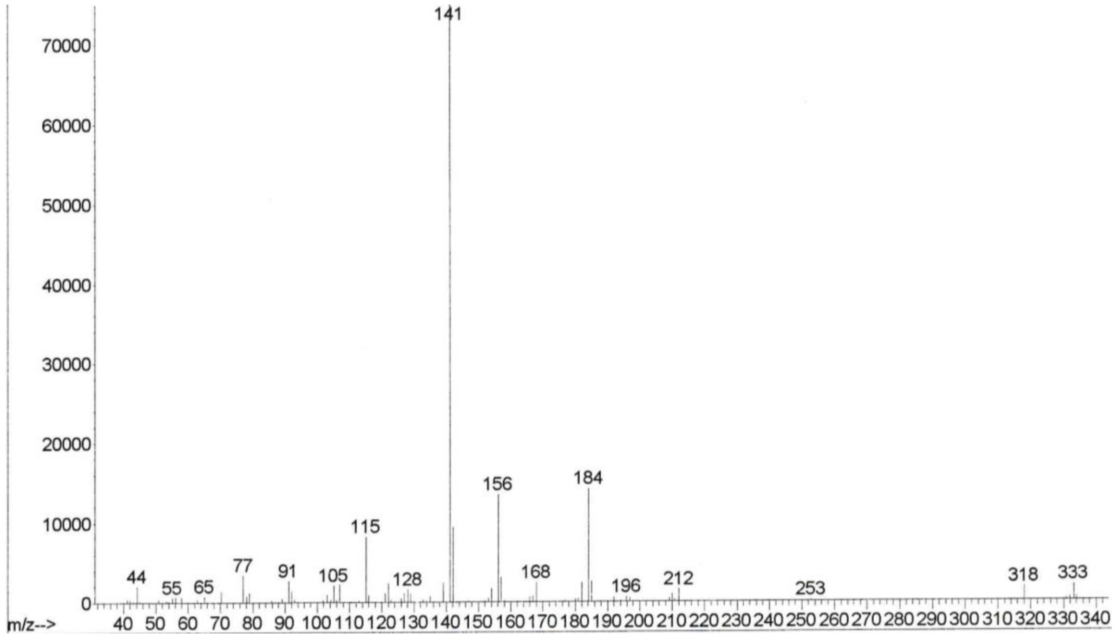

GC-MS (70 eV) of **17b**

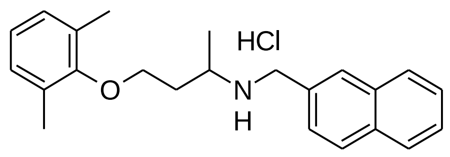

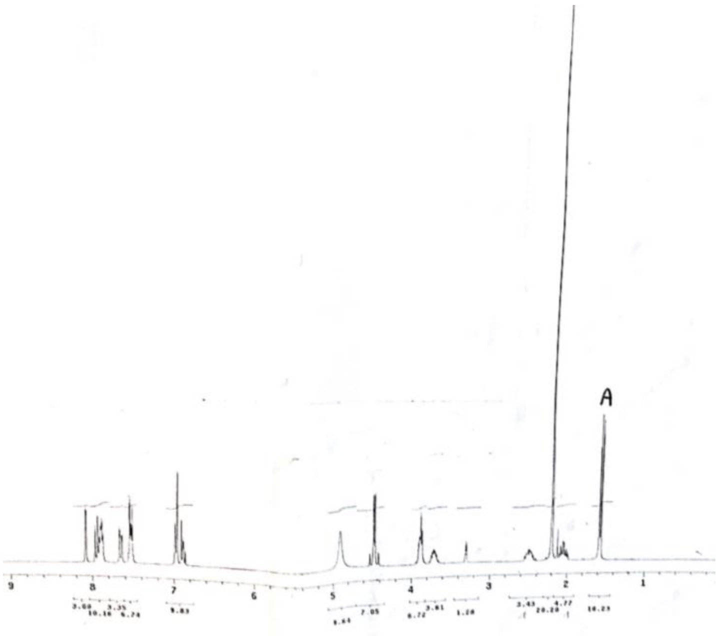

^{1}H NMR of **17b**·HCl (300 MHz, CD_3OD)

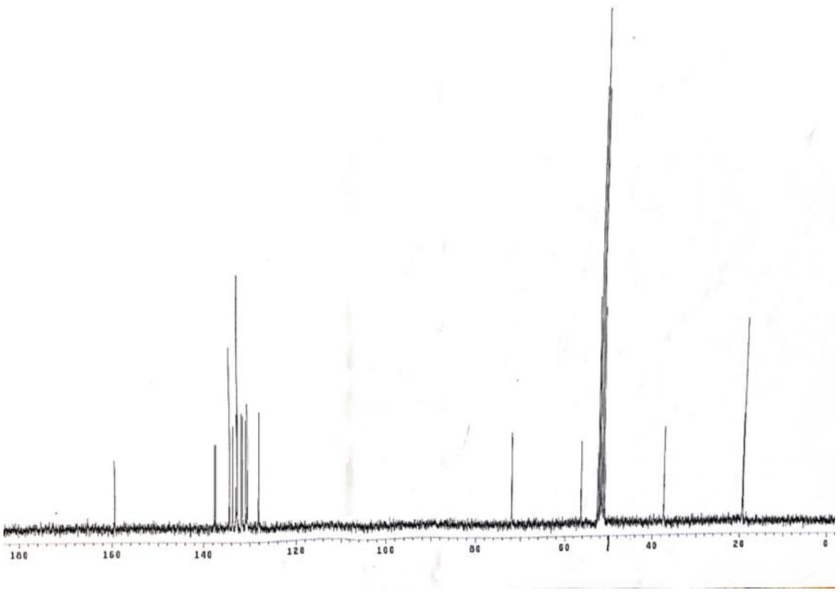

^{13}C NMR of **17b**·HCl (75 MHz, CD_3OD)

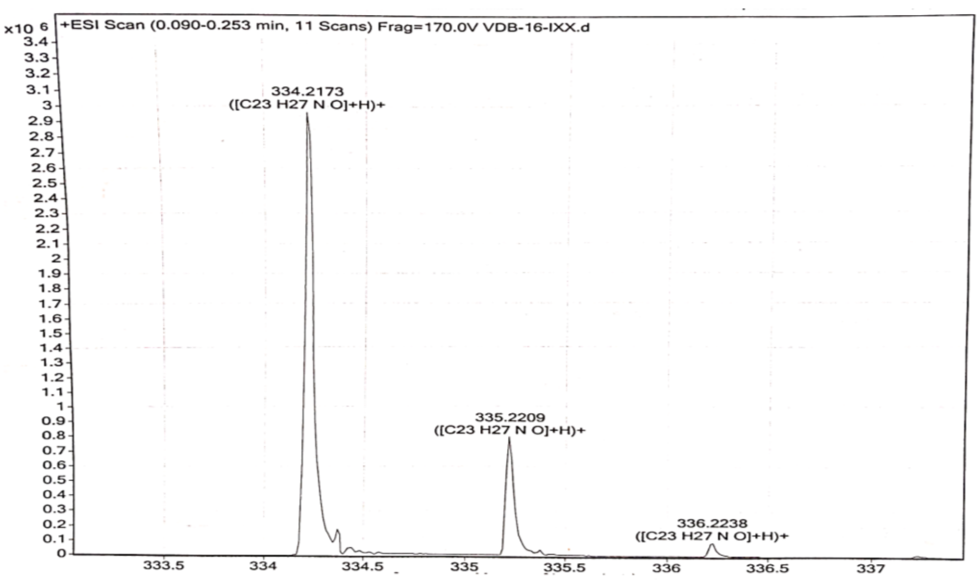

HRMS (QTOF) of **17b**·HCl

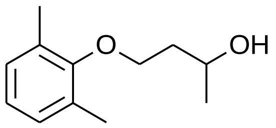

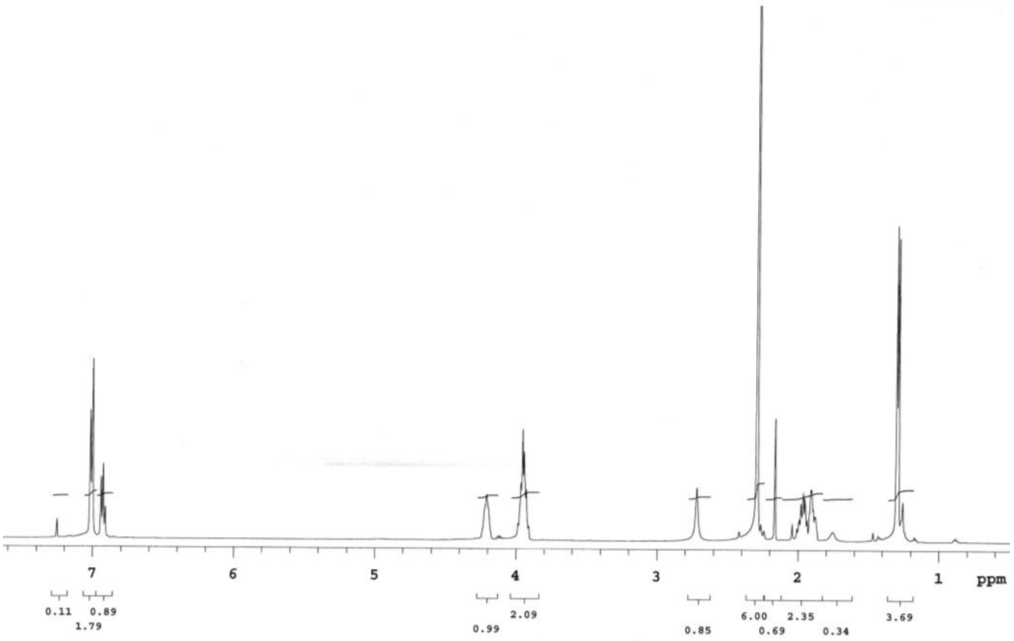

^{1}H NMR of **19** (500 MHz, $CDCl_3$)

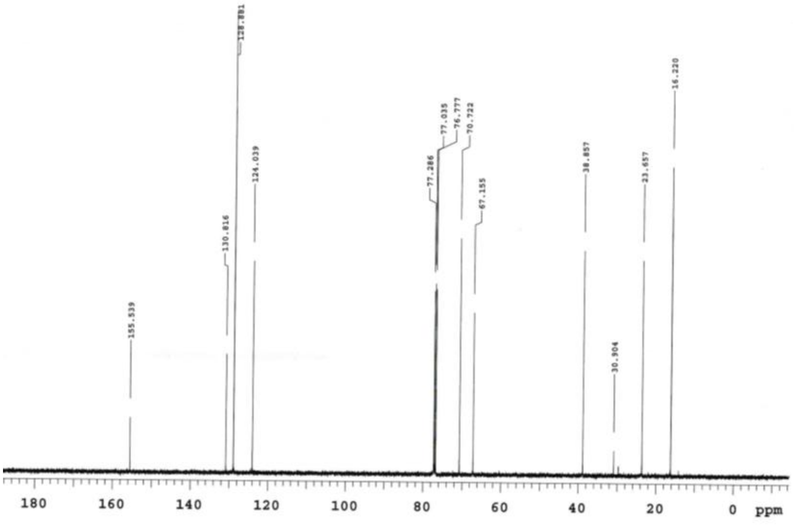

^{13}C NMR of **19** (125 MHz, $CDCl_3$)

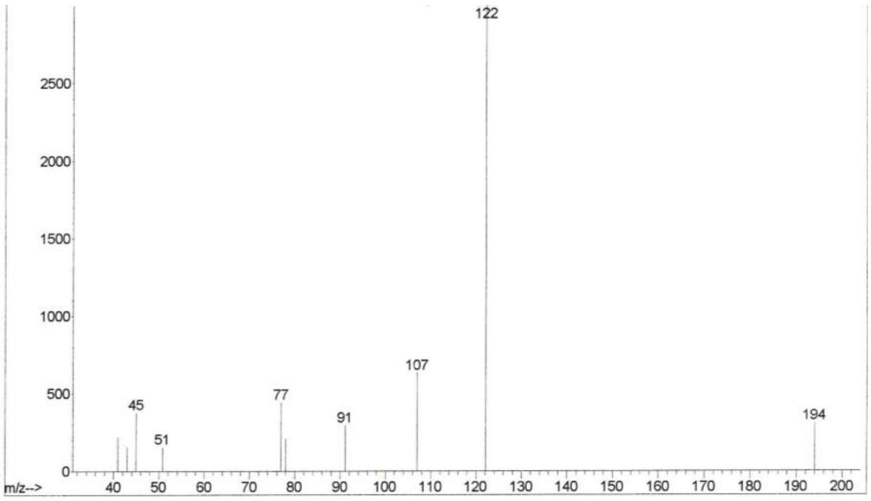

GC-MS (70 eV) of **19**

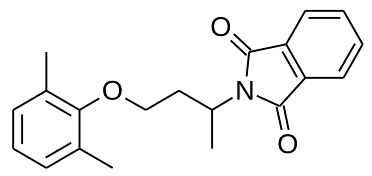

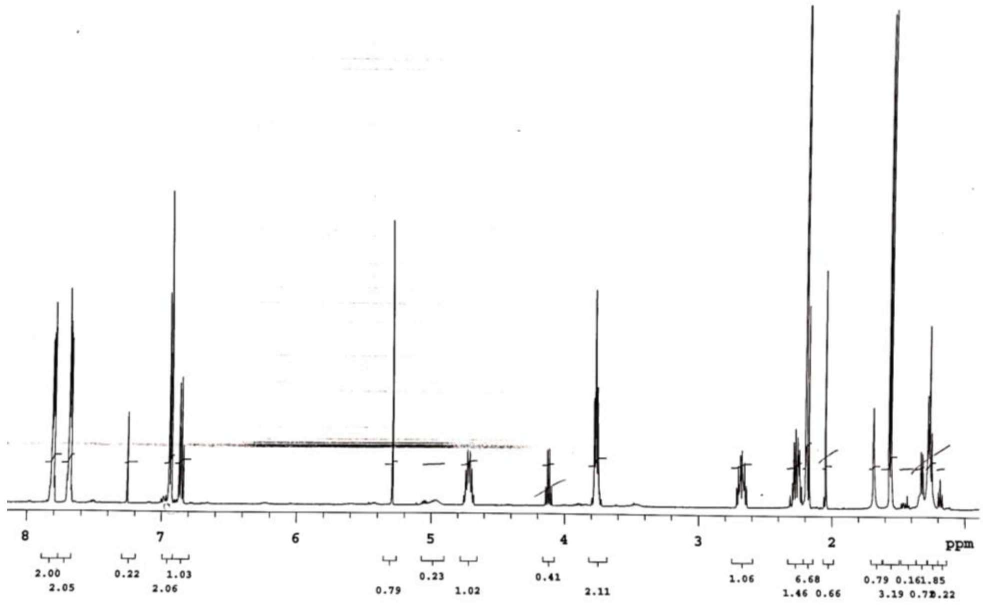

^{1}H NMR of **20** (500 MHz, $CDCl_3$)

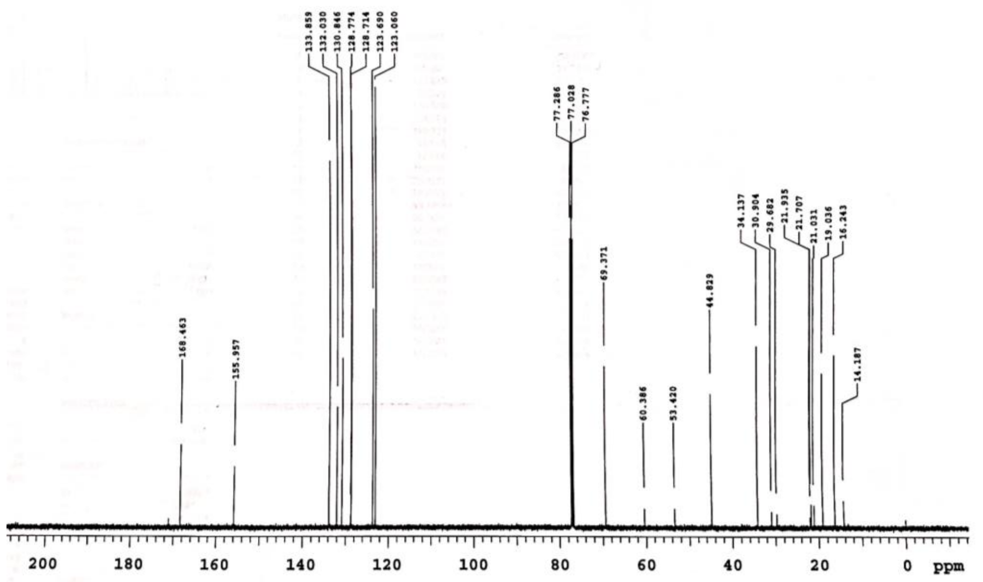

^{13}C NMR of **20** (125 MHz, $CDCl_3$)

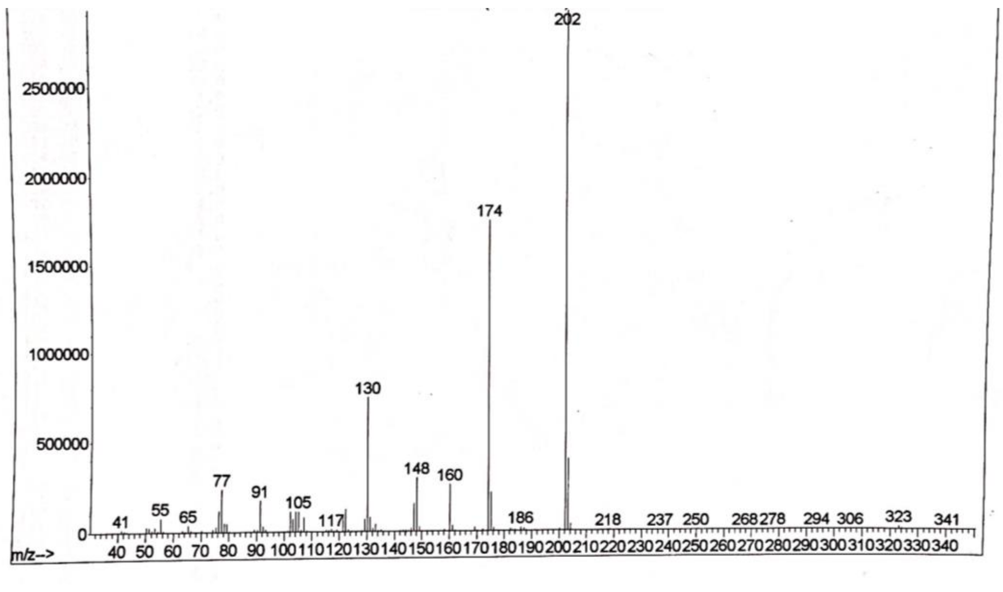

GC-MS (70 eV) of **20**

Table S1. ^{1}H NMR signal chemical shifts (ppm) in the aliphatic region of the **13a** spectrum

proton	chemical shift (ppm)		
	exptl		calcd[a]
	DMSO-d_6[1]	MeOD	
C*H*HCH	2.81 (dd)	2.99 (dd)	2.46 (dd)
CH*H*CH	3.10 (dd)	3.05 (dd)	2.74 (dd)
CH_2CH	3.84 (br s)	3.93 (m)	3.86 (m)
C*H*H	4.72 (br s)	4.80 (d)	4.65 (d)
CH*H*		4.86 (d)	4.99 (d)

[a]gaseous phase (DFT EDF2/6-31G*//DFT B3LYP/6-31G*); the reported chemical shifts were average values of the contribution of each conformer weighted through the corresponding Boltzmann distribution factor.

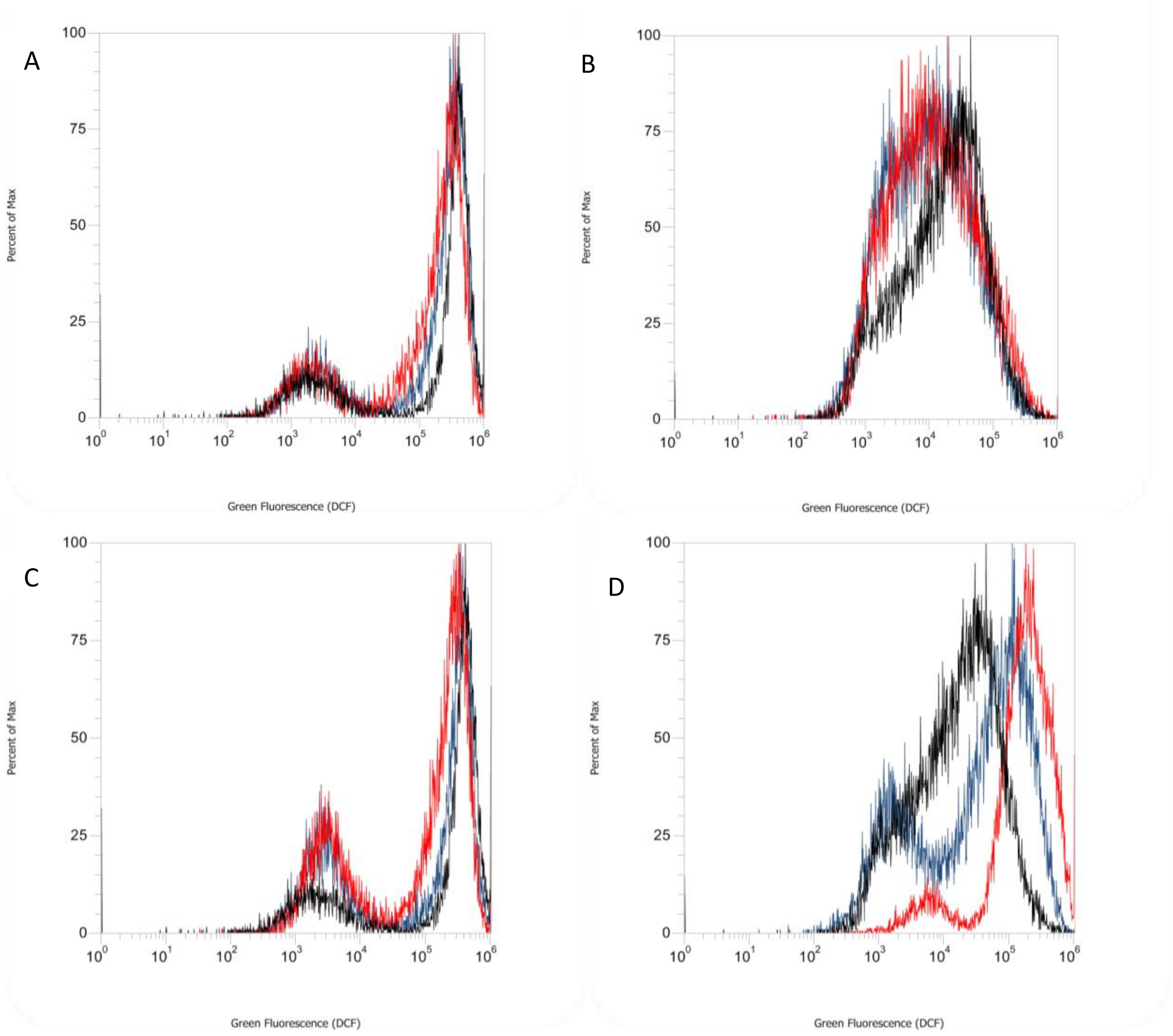

Figure S1: Flow cytometry analysis of ROS production by DCF-DA staining. HeLa cells were exposed to 1 and 50 μM of 13a (red line) and 17a (blue line) at 6 hr (Short term) (a,c) and 24 hr (Long term) (b, d). After washing with HBSS cells were stained with 5 μM of 2′,7′-dichorofluorescein diacetate (DCF-DA) for 30 min in dark. After washing, cells were resuspended in HBSS and analyzed using an Attune Acoustic Focusing Cytometer (Thermo Fisher Scientific) equipped with a 488 nm laser.

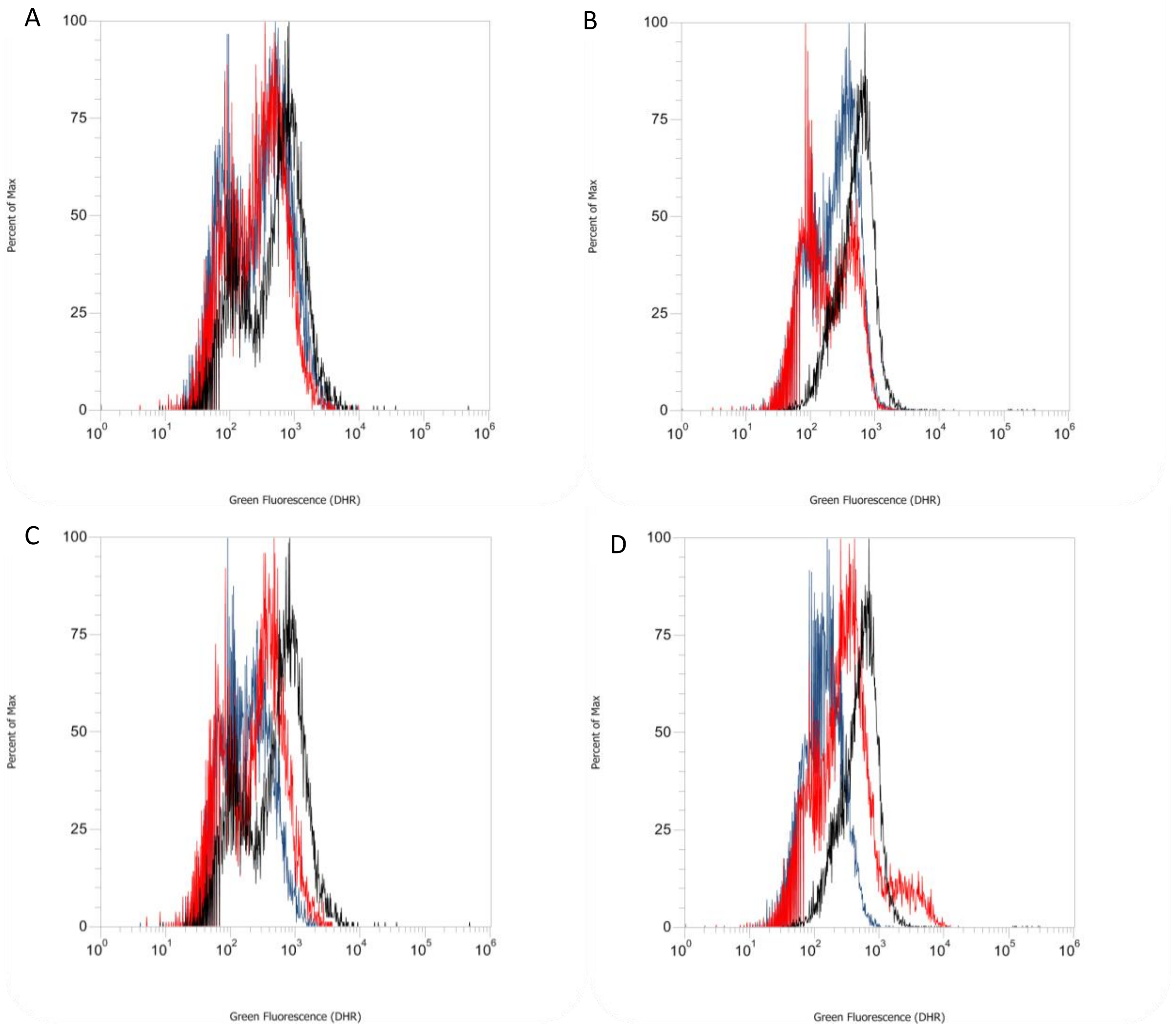

Figure S2: Flow cytometry analysis of ROS production by DHR staining. HeLa cells were exposed to 1 and 50 μM of 13a (red line) and 17a (blue line) at 6 hr (Short term) (a,c) and 24 hr (Long term) (b, d). After washing with HBSS cells were stained with 5 μM dihydrorhodamine (DHR) for 30 min in dark. After washing, cells were resuspended in HBSS and analyzed using an Attune Acoustic Focusing Cytometer (Thermo Fisher Scientific) equipped with a 488 nm laser.

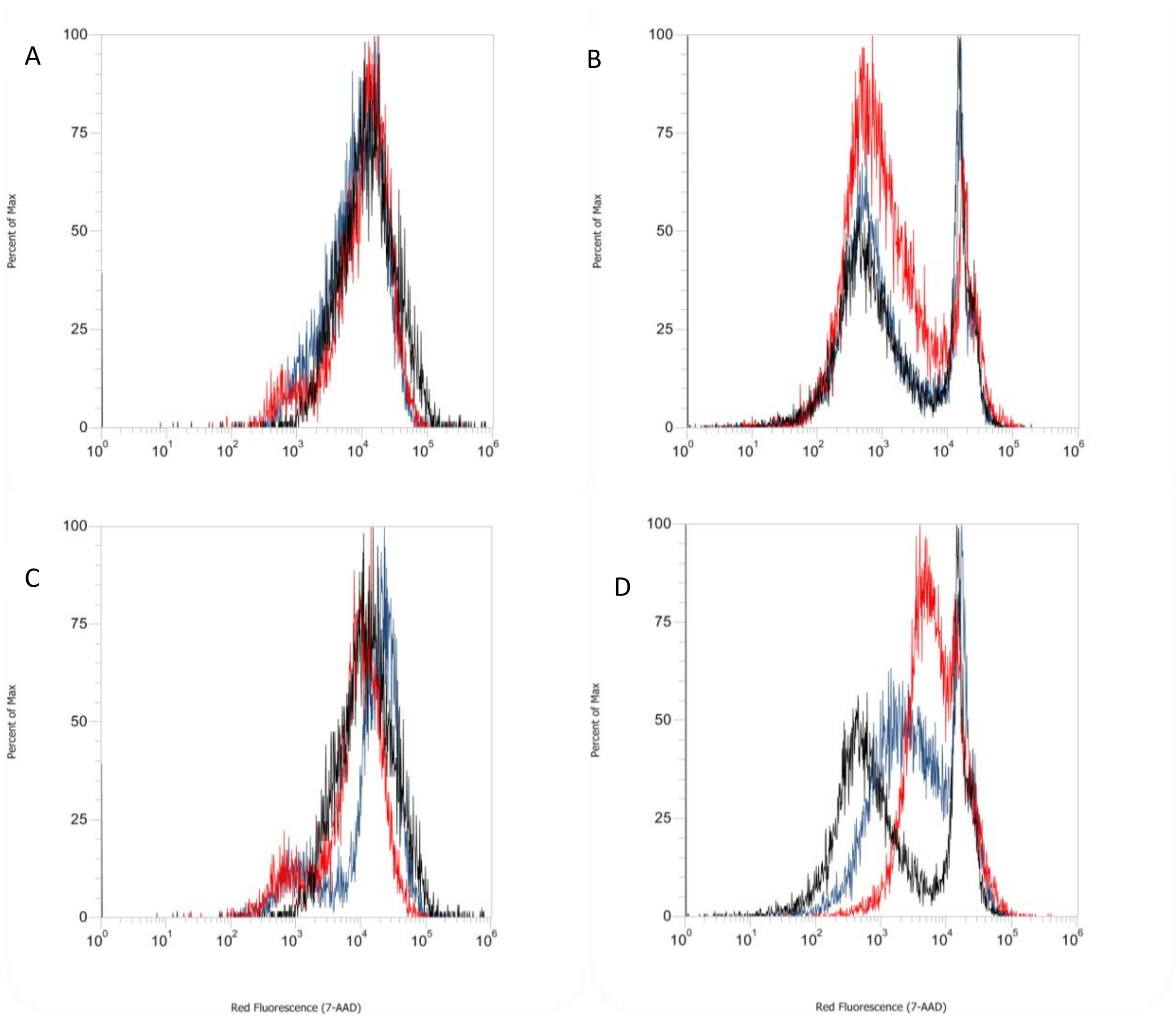

Figure S3: Flow cytometry analysis of cell viability ROS by DCF-DA and 7-AAD co-staining. HeLa cells were exposed to 1 and 50 μM of 13a (red line) and 17a (blue line) at 6 hr (Short term) (a,c) and 24 hr (Long term) (b, d). After washing with HBSS cells were stained with 5 μM of 2′,7′-dichorofluorescein diacetate (DCF-DA) for 30 min in dark. After washing, cells were resuspended in HBSS and analyzed using an Attune Acoustic Focusing Cytometer (Thermo Fisher Scientific) equipped with a 488 nm laser. Thereafter, DCFA-DA stained cells were co-stained with 1 μg/mL of 7-aminoactinomycin D (7-AAD), incubated on ice for 45 min in dark in order to asses viable and non-viable cells, and then flow cytometry analysis was carried out.

Reference

1. M. Muraglia, M. De Bellis, A. Catalano, A. Carocci, C. Franchini, A. Carrieri, C. Fortugno, C. Bertucci, J.-F. Desaphy, A. De Luca, D. Conte Camerino, F. Corbo *N*-aryl-2,6-dimethylbenzamides, a new generation of tocainide analogues as blockers of skeletal muscle voltage-gated sodium channels. J. Med. Chem 57 (2014) 2589–2600.
